# Supplementary material for: DNA methylation-environment interactions in the human genome
Source: bioRxiv. 2023 Dec 15:2023.05.19.541437. Originally published 2023 May 19. Preprint. [Version 2] doi: 10.1101/2023.05.19.541437 (PMC10245841; doi:10.1101/2023.05.19.541437)
Supplement: 1 — Figure 1-figure supplement 1. Overlap of target genomic regions with each other. Figure 1-figure supplement 2. mSTARR-seq library diversity. Figure 1-figure supplement 3. Methylation levels of mSTARR-seq DNA, pre- and posttransfection. Figure 1-figure supplement 4. Rarefaction curve showing total number of windows formally tested for regulatory activity, as a function of number of reads sequenced per DNA or RNA replicate. Figure 1-figure supplement 5. Overlap of regulatory activity across datasets. Figure 1-figure supplement 6. Correlations between RNA and DNA replicates. Figure 1-figure supplement 7. Library diversity and regions of regulatory activity in HepG2 cells. Figure 1-figure supplement 8. Methylation-dependent regulatory activity across datasets. Figure 1-figure supplement 9. RNA to DNA ratios in the methylated and unmethylated replicates in the baseline dataset. Figure 1-figure supplement 10. Histograms of RNA to DNA ratios in the baseline dataset. Figure 1-figure supplement 11. Relationship between CpG density and methylation-dependent regulatory activity. Figure 2-figure supplement 1. Filtering results across datasets. Figure 2-figure supplement 2. Overlap of tested genomic windows across datasets. Figure 2-figure supplement 3. Overlap of regulatory activity and effects of methylation across environmental conditions. Figure 2-figure supplement 4. IFIT5 endogenous gene expression is responsive to IFNA stimulation. Figure 3-figure supplement 1. Across individuals, methylation in the mSTARR-seq annotated enhancer chr1:1013400–1014000 predicts the ISG15 gene expression response to flu. Figure 4-figure supplement 1. Summary of early-life adversity (ELA) studies. [file NIHPP2023.05.19.541437V2-supplement-1.pdf]

bioRxiv preprint doi: https://doi.org/10.1101/2023.05.19.541437; this version posted December 15, 2023. The copyright holder for this preprint (which was not certified by peer review) is the author/funder, who has granted bioRxiv a license to display the preprint in perpetuity. It is made available under aCC-BY 4.0 International license.

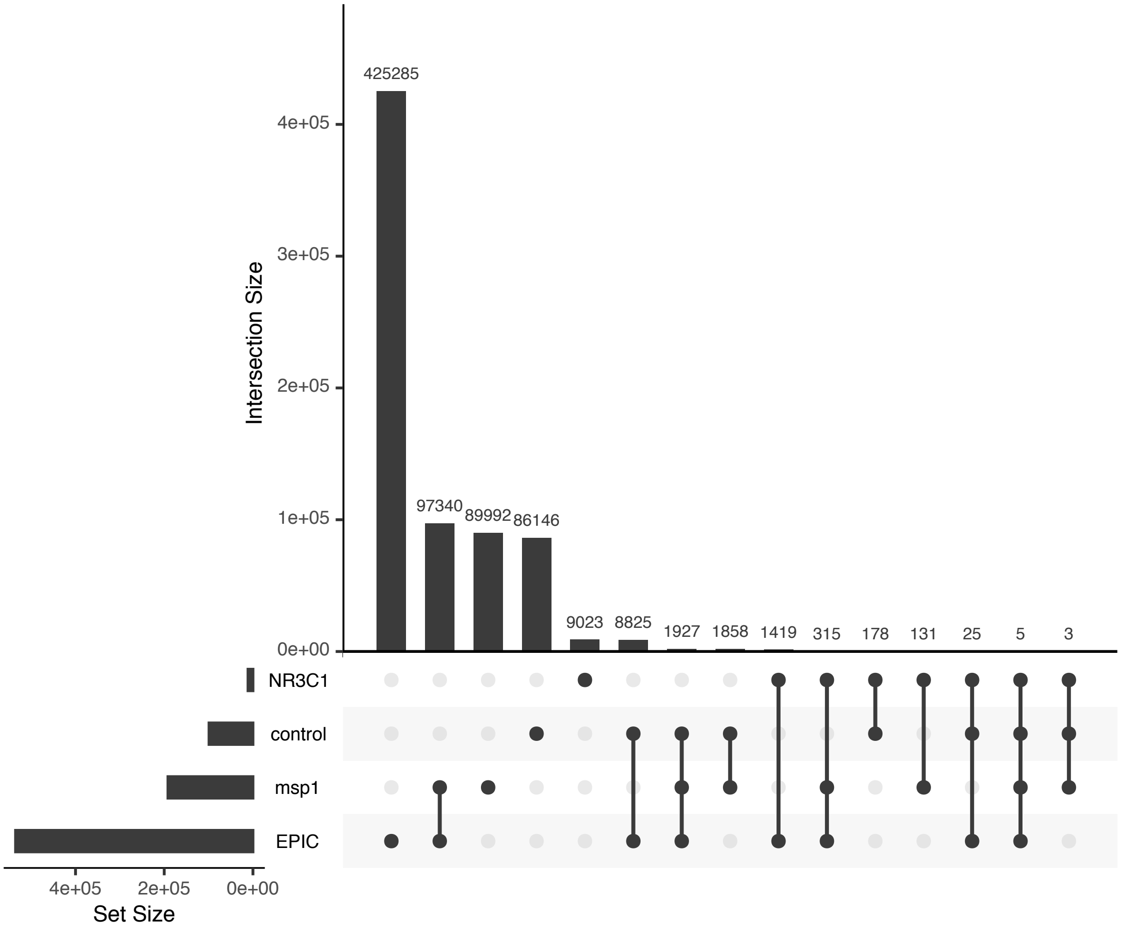

Figure 1-figure supplement 1. Overlap of target genomic regions with each other. Upset plot showing the degree to which 600 bp non-overlapping genomic windows are shared between the four target genomic regions (EPIC CpGs, *Msp1* CpG cut sites, the *NR3C1* region, or control sites). Overlap occurs because a single 600 bp genomic window can simultaneously include EPIC CpGs, *Msp1* CpG cut sites, the *NR3C1* region, and/or control sites. This plot includes 722,472 unique windows, reflecting the set of windows containing at least 1 basepair of sequence in the target loci.

bioRxiv preprint doi: https://doi.org/10.1101/2023.05.19.541437; this version posted December 15, 2023. The copyright holder for this preprint (which was not certified by peer review) is the author/funder, who has granted bioRxiv a license to display the preprint in perpetuity. It is made available under aCC-BY 4.0 International license.

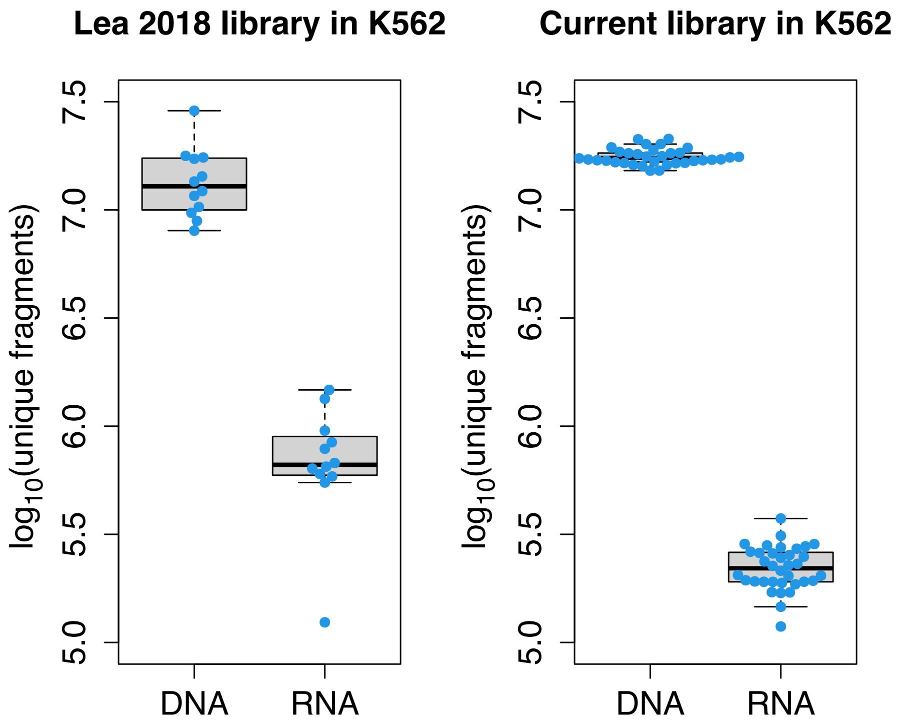

Figure 1-figure supplement 2. mSTARR-seq library diversity. Comparison of diversity of unique mSTARR-seq DNA and RNA fragments from the library generated in this study (transfected into K562 cells) relative to the library published in Lea *et al*., 2018 (independently transfected into K562 cells). Each dot represents an experimental replicate. Each box represents the interquartile range, with the median value depicted as a horizontal bar. Whiskers extend to the most extreme values within 1.5x of the interquartile range.

bioRxiv preprint doi: https://doi.org/10.1101/2023.05.19.541437; this version posted December 15, 2023. The copyright holder for this preprint (which was not certified by peer review) is the author/funder, who has granted bioRxiv a license to display the preprint in perpetuity. It is made available under aCC-BY 4.0 International license.

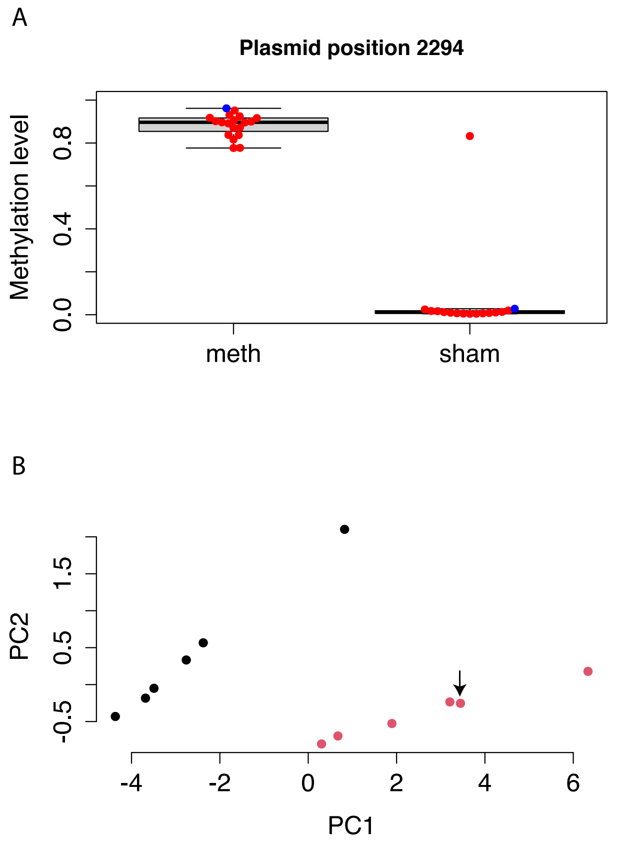

Figure 1-figure supplement 3. Methylation levels of mSTARR-seq DNA, pre- and post-transfection. (A) Bisulfite sequencing shows that DNA methylation on the mSTARR-seq plasmid is maintained until the end of the experiment (i.e., 48 hours after transfection), with significantly higher methylation levels in the replicates from the methyltransferase reaction relative to the replicates from the sham methyltransferase reaction (mean methylated = 0.885, mean unmethylated = 0.066; unpaired t-test: t = -14.66, df = 15,124, p = 2.39 x 10^{-10}). Each dot represents an experimental replicate. Red dots indicate post-transfection DNA samples; the single blue dot per condition indicates pre-transfection DNA methylation levels. Methylation estimates are based on the CpG at the position 2294, which is located in the plasmid region used for Gibson assembly. We assessed methylation of this CpG, rather than across CpGs genome-wide, because the genomic coverage of our bisulfite sequencing data across replicates was too variable to perform reliable site-by-site analysis of DNA methylation levels before and after the 48 hour experiment. One sample from the dex sham reaction, L31395, shows an unexpectedly high level of methylation, which appears to be due to an error during generation of the bisulfite sequencing library (e.g., mislabeled tube or poor bisulfite conversion), and not the experimental replicate of cells itself, as the mSTARR-seq RNA library (L31244) from the same replicate clusters with the unmethylated sham replicates as expected (panel B). **(B)** The first two principal components summarizing overall counts of mSTARR-seq reads for the dex-treated RNA samples (i.e., the raw readout of overall regulatory activity). Each dot represents an experimental replicate, with red and black indicating sham and methylated replicates, respectively. Overall regulatory activity of sample L31244 (indicated by arrow) clusters with the sham replicates as expected, suggesting that this replicate was indeed transfected with sham-treated mSTARR-seq DNA.

bioRxiv preprint doi: https://doi.org/10.1101/2023.05.19.541437; this version posted December 15, 2023. The copyright holder for this preprint (which was not certified by peer review) is the author/funder, who has granted bioRxiv a license to display the preprint in perpetuity. It is made available under aCC-BY 4.0 International license.

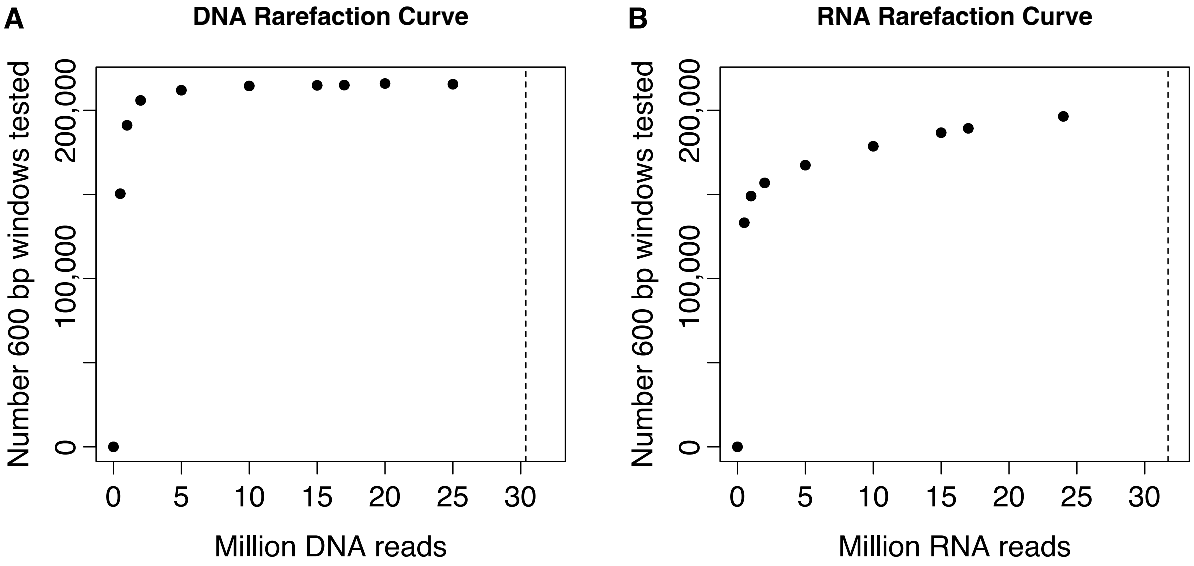

Figure 1-figure supplement 4. Rarefaction curve showing total number of windows formally tested for regulatory activity, as a function of number of reads sequenced per DNA or RNA replicate. Sequencing reads from the (A) DNA replicates or (B) RNA replicates of the baseline dataset were rarefied to the values shown on the x-axis before running the data processing steps and applying the filtering criteria described in the Materials and Methods for the full data set. Dashed vertical lines represent the mean number of sequenced reads per DNA replicate (mean [SD] = 30.375 million [3.335 million]) or RNA replicate (mean [SD] = 31.712 million [8.194 million]) in the full baseline dataset. These analyses show that our sequencing effort saturated the number of formally analyzable windows based on either our criteria for inclusion based on DNA library sequencing depth or RNA library sequencing depth.

bioRxiv preprint doi: https://doi.org/10.1101/2023.05.19.541437; this version posted December 15, 2023. The copyright holder for this preprint (which was not certified by peer review) is the author/funder, who has granted bioRxiv a license to display the preprint in perpetuity. It is made available under aCC-BY 4.0 International license.

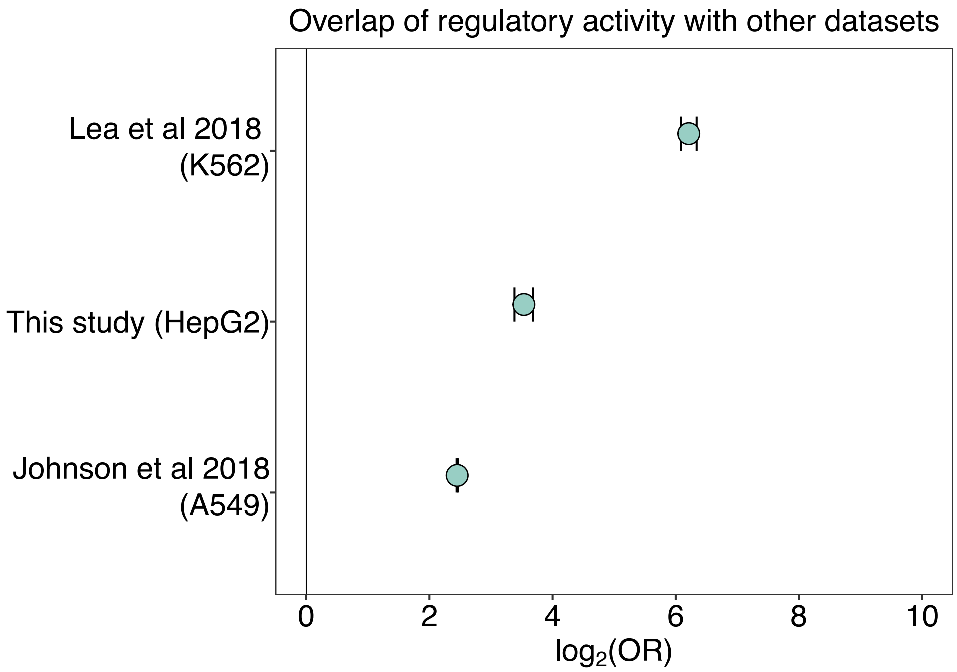

Figure 1-figure supplement 5. Overlap of regulatory activity across datasets. Regulatory regions (in either the unmethylated sham condition, the methylated condition, or both) identified via mSTARR-seq in this study significantly overlap with: K562 regulatory regions (in either the unmethylated sham or methylated condition, or both) from a previously generated mSTARR-seq dataset reanalyzed with our pipeline (Lea et al., 2018) ($\log_2$(OR) [95% CI] = 6.212 [6.086, 6.440], $p < 1.0 \times 10^{-300}$); regulatory regions (in either the unmethylated sham or methylated condition, or both) from an mSTARR-seq experiment in HepG2 liver cells ($\log_2$(OR) [95% CI] = 3.534 [3.381, 3.684], $p = 5.21 \times 10^{-307}$); and regulatory regions from a conventional STARR-seq experiment (i.e., an unmethylated condition) in A549 lung epithelial cells (Johnson et al., 2018) ($\log_2$(OR) [95% CI] = 2.451 [2.442, 2.461], $p < 1.0 \times 10^{-300}$). Bars represent 95% confidence intervals.

bioRxiv preprint doi: https://doi.org/10.1101/2023.05.19.541437; this version posted December 15, 2023. The copyright holder for this preprint (which was not certified by peer review) is the author/funder, who has granted bioRxiv a license to display the preprint in perpetuity. It is made available under aCC-BY 4.0 International license.

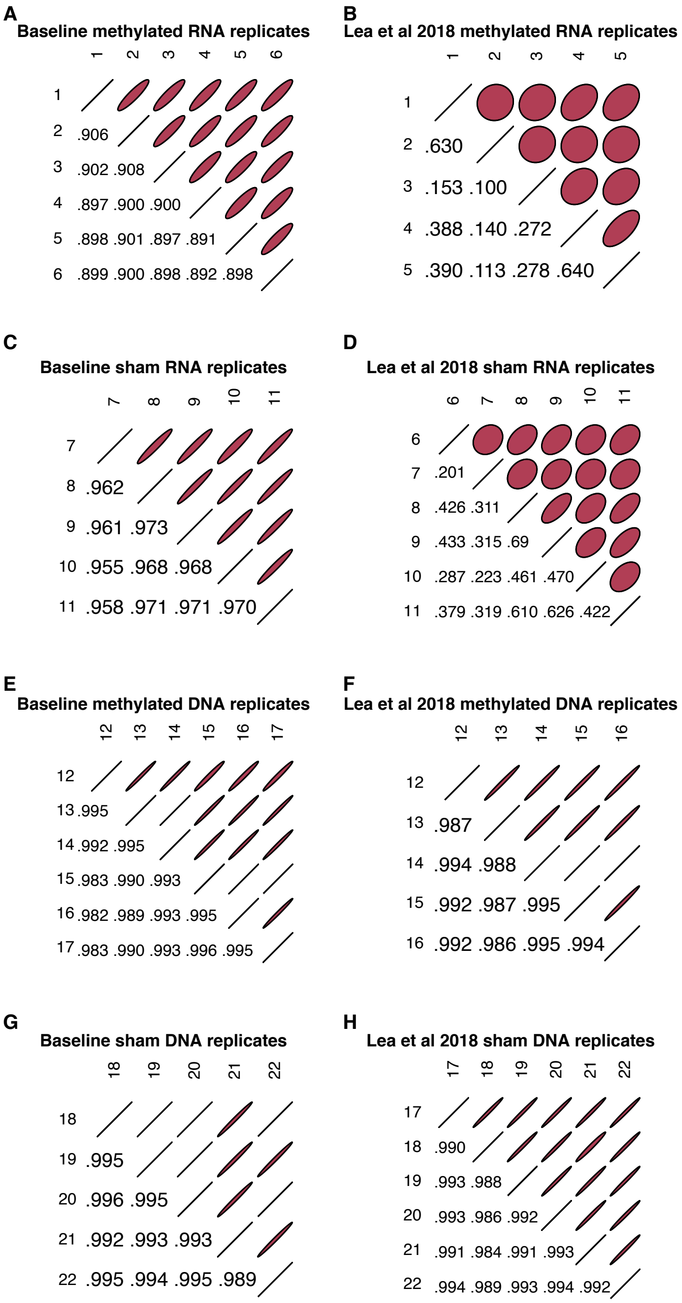

Figure 1-figure supplement 6. Correlations between RNA and DNA replicates. Pearson correlations (r) of raw counts between RNA replicates (A-D) and between DNA replicates (E-H) within the windows we formally analyzed for enhancer activity in the baseline dataset reported here and in Lea *et al*., 2018, following a uniform data processing pipeline. All replicate pairs (both RNA and DNA, in both sham and methylated conditions) in the baseline dataset show correlations ≥ 0.89, demonstrating replicate reproducibility comparable to other STARR-seq studies (e.g., Klein et al., 2020). For RNA libraries, replicates in the baseline dataset are more correlated than in the Lea *et al*., 2018 dataset (RNA replicates: baseline mean r = 0.926; Lea *et al*., 2018 mean r = 0.347), although DNA replicates show similar inter-replicate consistency (baseline mean r = 0.992; *Lea et al*., 2018 mean r = 0.991).

bioRxiv preprint doi: https://doi.org/10.1101/2023.05.19.541437; this version posted December 15, 2023. The copyright holder for this preprint (which was not certified by peer review) is the author/funder, who has granted bioRxiv a license to display the preprint in perpetuity. It is made available under aCC-BY 4.0 International license.

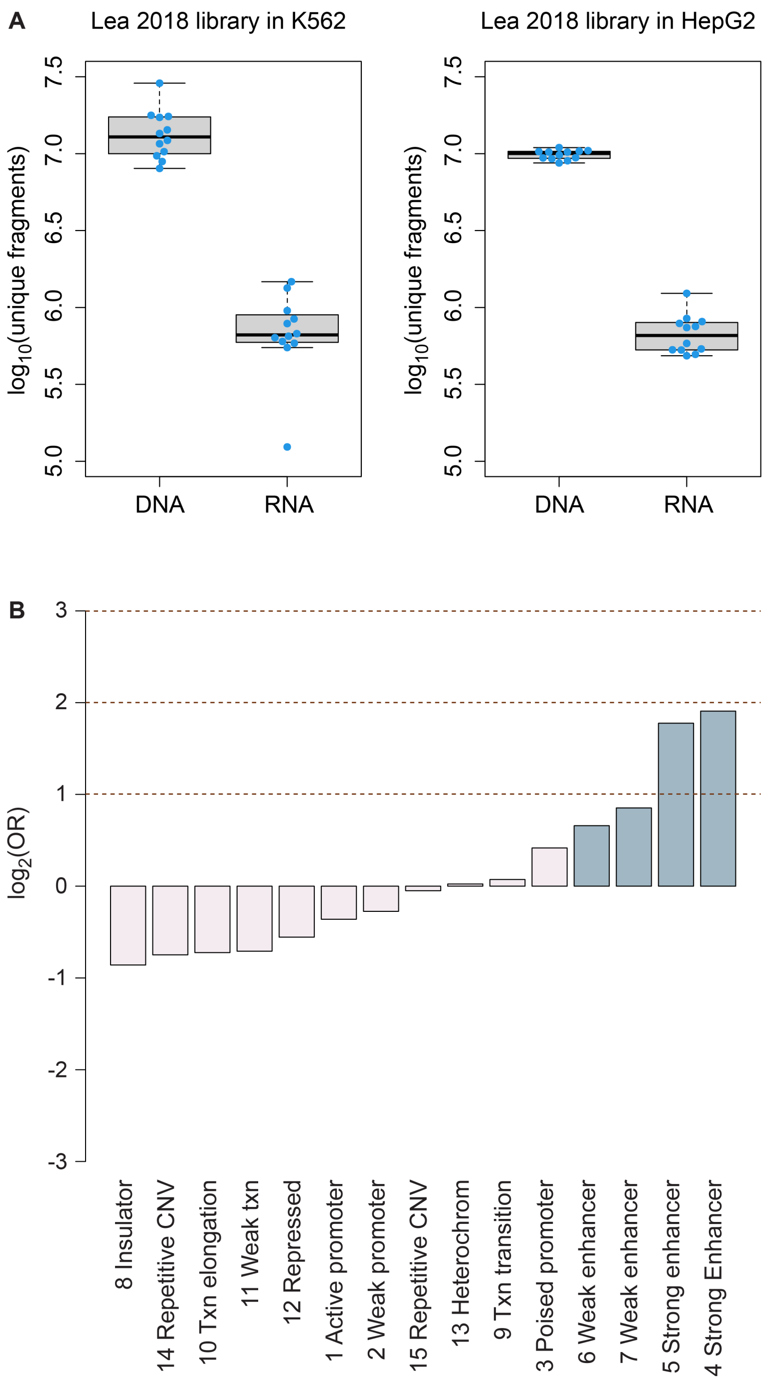

Figure 1-figure supplement 7. Library diversity and regions of regulatory activity in HepG2 cells. (A) Comparison of diversity of unique mSTARR-seq DNA and RNA fragments from the library published in Lea *et al.*, 2018 (transfected into K562 cells) versus the same library transfected into HepG2 cells in this study. Each dot represents an experimental replicate. Each box represents the interquartile range, with the median value depicted as a horizontal bar. Whiskers extend to the most extreme values within 1.5x of the interquartile range. **(B)** mSTARR-seq regulatory activity in HepG2 cells is strongly enriched in ENCODE-defined enhancers (indicated in blue) and some classes of promoters, and depleted in repressed and repetitive states.

bioRxiv preprint doi: https://doi.org/10.1101/2023.05.19.541437; this version posted December 15, 2023. The copyright holder for this preprint (which was not certified by peer review) is the author/funder, who has granted bioRxiv a license to display the preprint in perpetuity. It is made available under aCC-BY 4.0 International license.

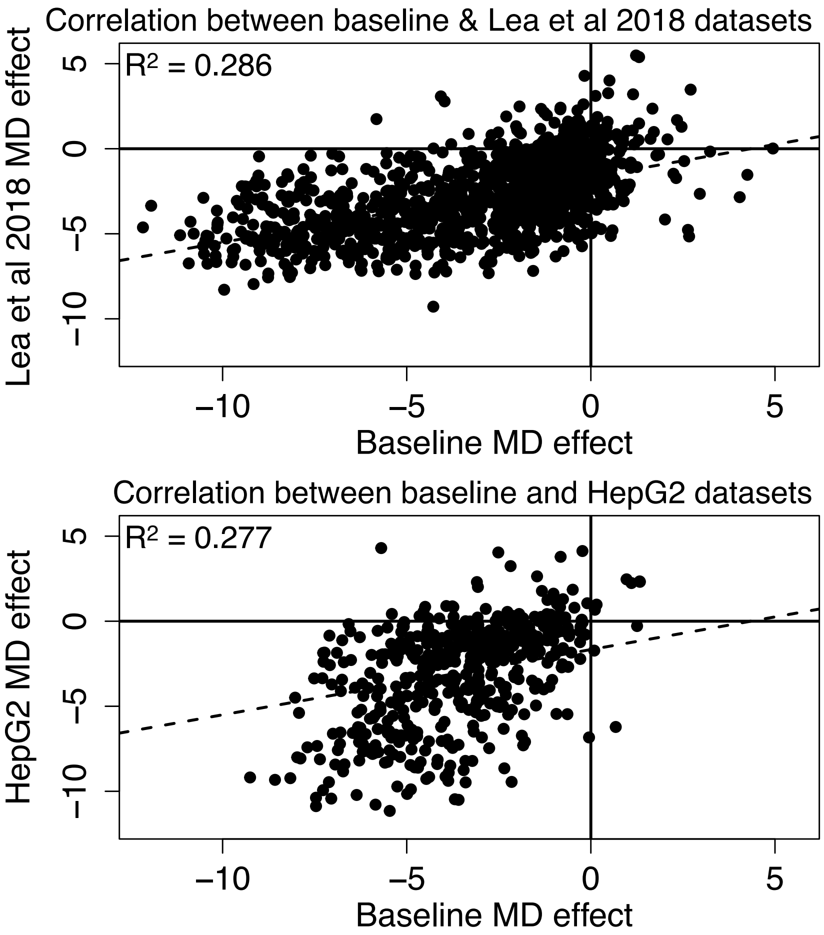

Figure 1-figure supplement 8. Methylation-dependent regulatory activity across datasets. Effects of methylation on regulatory activity estimated in this study in the baseline dataset are consistent with methylation effects in K562s estimated from a previously generated mSTARR-seq dataset (Lea *et al.*, 2018) and with methylation effects estimated in HepG2 liver cells (Lea *et al.*, 2018: Pearson's r = 0.534 for 1250 windows with FDR < 1% in both data sets, $R^2 = 0.286$, p = 3.19 x 10^{-93}; HepG2: Pearson's r = 0.526 for 511 windows with FDR < 1% in both data sets, $R^2 = 0.277$, p = 8.87 x 10^{-38}). Each dot represents a 600 bp regulatory window identified, in either the sham or methylated states, in both datasets (FDR < 1%; note that not all regulatory windows show significant methylation dependence [MD]). Dashed lines are the best fit lines. In all cases, negative effect sizes correspond to reduced activity in the methylated condition and positive effect sizes correspond to increased activity in the methylated condition.

bioRxiv preprint doi: https://doi.org/10.1101/2023.05.19.541437; this version posted December 15, 2023. The copyright holder for this preprint (which was not certified by peer review) is the author/funder, who has granted bioRxiv a license to display the preprint in perpetuity. It is made available under aCC-BY 4.0 International license.

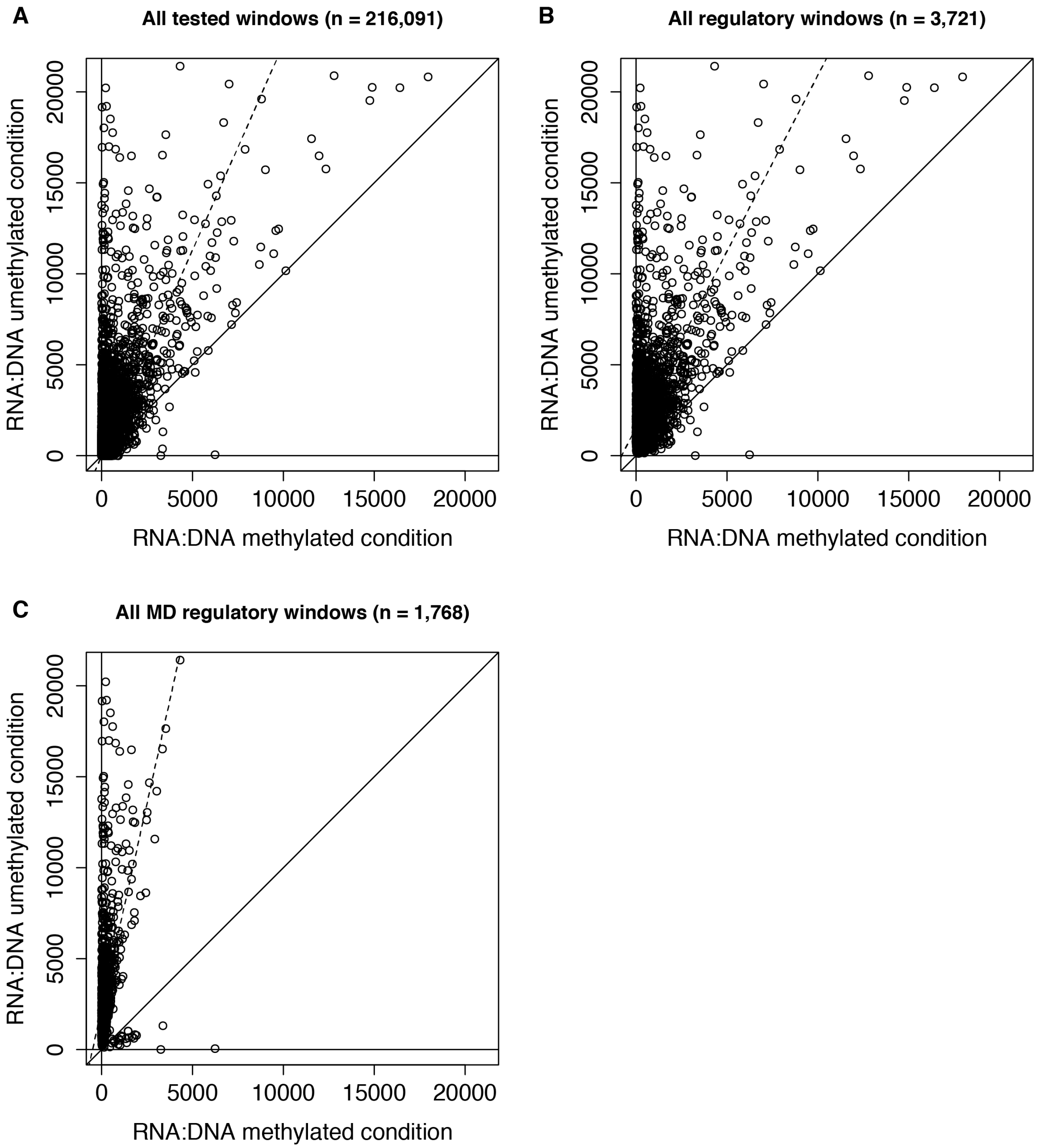

Figure 1-figure supplement 9. RNA to DNA ratios in the methylated and unmethylated replicates in the baseline dataset. Mean RNA (in counts per million) to DNA ratios formethylated replicates (x-axis) versus unmethylated replicates (y-axis). A constant of 0.5 was added to the initial raw counts to ensure no denominator values were 0. Each dot represents a 600 bp window that was formally tested for enhancer activity (**A**), exhibited significant regulatory activity (**B**), or exhibited significant methylation-dependent regulatory activity (**C**) in the baseline dataset. Solid diagonal lines represent y = x, and dashed lines represent the best fit lines. As expected, 600 bp windows tend to show higher RNA to DNA ratios in the unmethylated condition relative to the methylated condition.

bioRxiv preprint doi: https://doi.org/10.1101/2023.05.19.541437; this version posted December 15, 2023. The copyright holder for this preprint (which was not certified by peer review) is the author/funder, who has granted bioRxiv a license to display the preprint in perpetuity. It is made available under aCC-BY 4.0 International license.

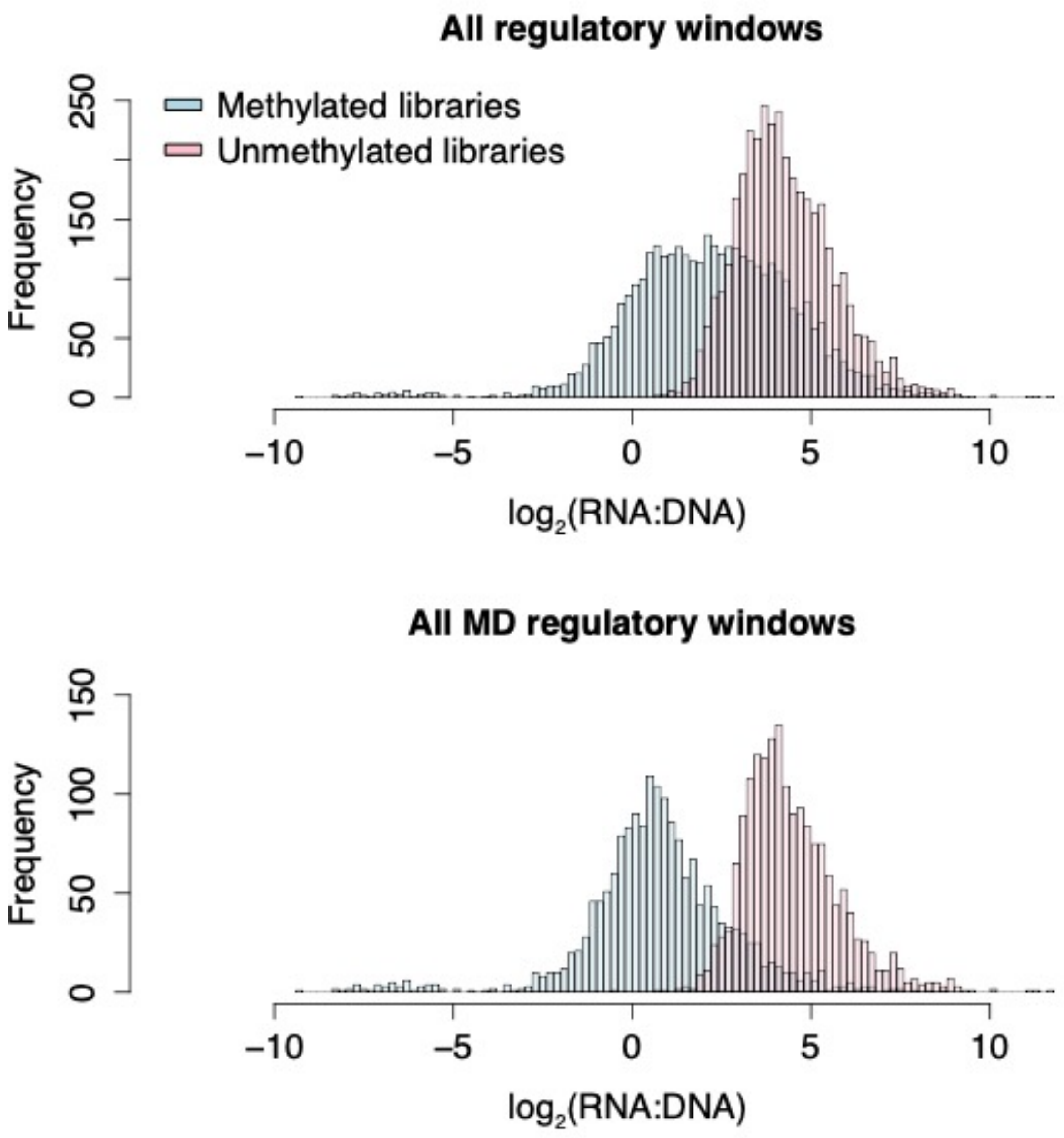

Figure 1-figure supplement 10. Histograms of RNA to DNA ratios in the baseline dataset. The x-axis represents the $\log_2$(mean RNA [in counts per million] to DNA ratios) for the baseline dataset. A constant of 0.5 was added to the initial raw counts to prevent 0 from being in the denominator. Lower values on the x-axis indicate windows showing lower regulatory activity.

bioRxiv preprint doi: https://doi.org/10.1101/2023.05.19.541437; this version posted December 15, 2023. The copyright holder for this preprint (which was not certified by peer review) is the author/funder, who has granted bioRxiv a license to display the preprint in perpetuity. It is made available under aCC-BY 4.0 International license.

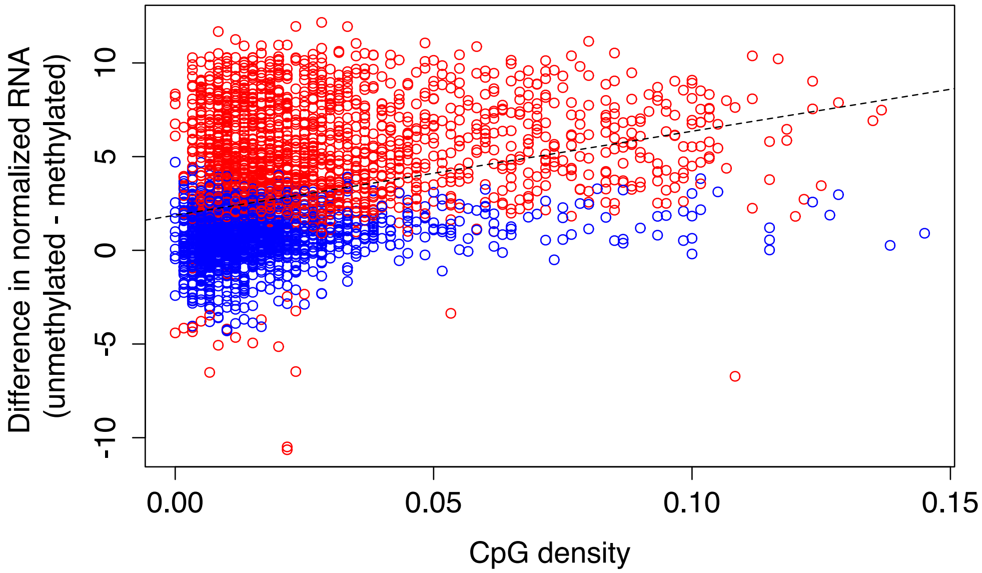

Figure 1-figure supplement 11. Relationship between CpG density and methylation-dependent regulatory activity. CpG-dense mSTARR-seq regulatory regions are more likely to be repressed by DNA methylation (positive y-axis value; Spearman's rho = 0.370, p = 9.865 x 10^{-121}; n = 3,721 regions with mSTARR-seq regulatory activity). Each dot represents a 600 bp window that showed significant regulatory activity (FDR < 1%). Red and blue dots represent regulatory windows where methylation-dependent activity was or was not detected, respectively. The dashed line represents the best fit line.

bioRxiv preprint doi: https://doi.org/10.1101/2023.05.19.541437; this version posted December 15, 2023. The copyright holder for this preprint (which was not certified by peer review) is the author/funder, who has granted bioRxiv a license to display the preprint in perpetuity. It is made available under aCC-BY 4.0 International license.

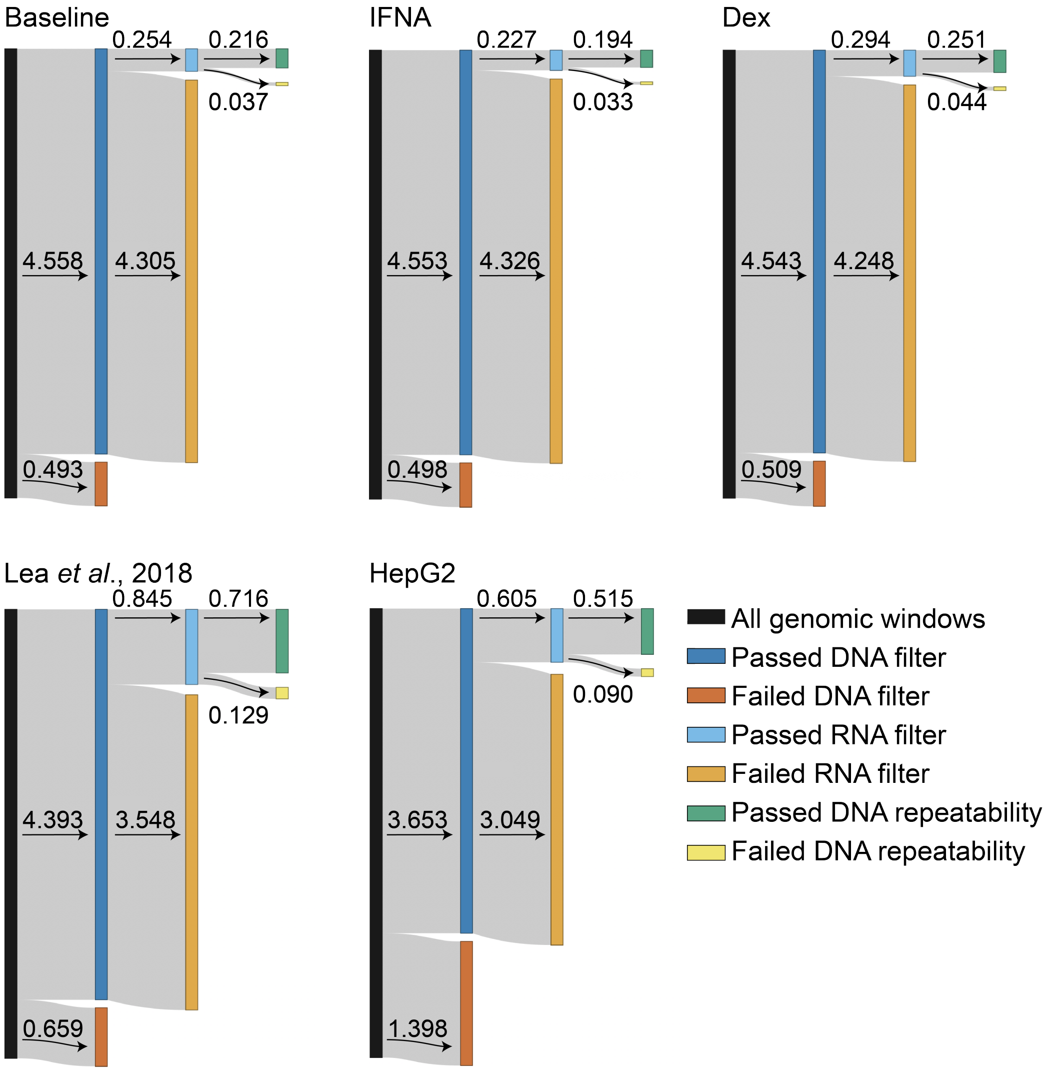

Figure 2-figure supplement 1. Filtering results across datasets. Each of the 5 datasets began with 5,051,776 600 bp genomic windows. For each of the 5 datasets, we reduced the dataset to windows that had nonzero counts in at least three DNA samples in the methylated condition *and* three DNA samples in the unmethylated condition (i.e., 6 DNA samples total; "DNA filter"). We then reduced the dataset to windows that had nonzero counts in at least three RNA samples in either the methylated *or* unmethylated condition ("RNA filter"). Finally, we retained only windows that showed high repeatability across DNA samples, following (Lea *et al.*, 2018) ("DNA repeatability"). Numbers correspond to million windows that passed or failed each filter for which the arrow points to. Note that one mSTARR RNA-seq sample in the baseline condition [sample ID L31250] was removed from further analysis because it had an unusually high proportion of zero counts in the testable windows; we therefore also removed the corresponding paired DNA sample prior to analysis). See Supplementary File 9 for the precise window numbers corresponding to the plots.

bioRxiv preprint doi: https://doi.org/10.1101/2023.05.19.541437; this version posted December 15, 2023. The copyright holder for this preprint (which was not certified by peer review) is the author/funder, who has granted bioRxiv a license to display the preprint in perpetuity. It is made available under aCC-BY 4.0 International license.

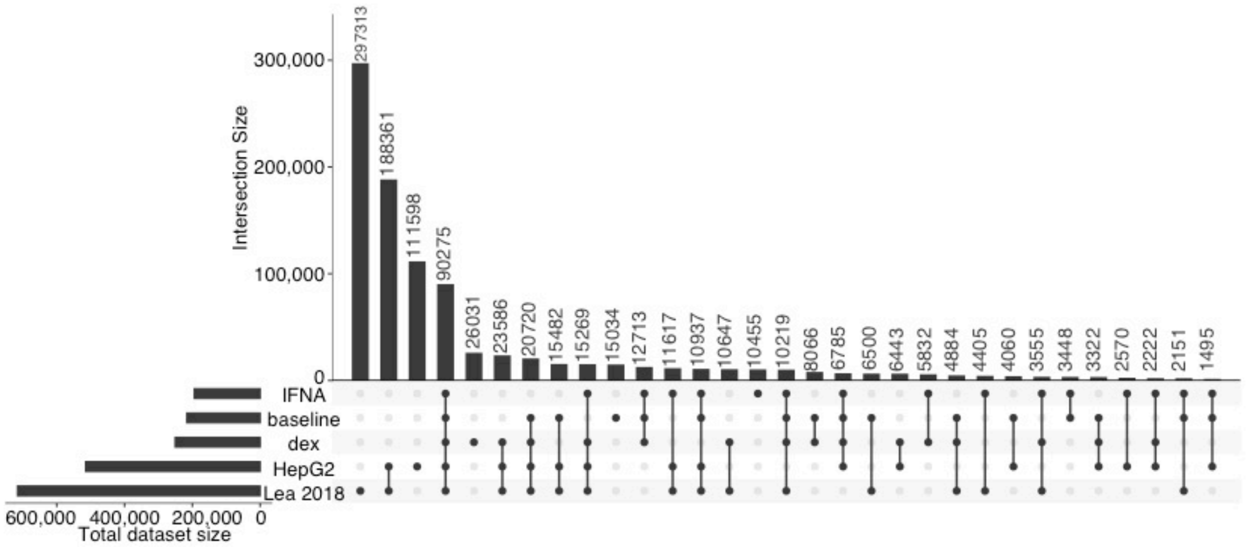

Figure 2-figure supplement 2. Overlap of tested genomic windows across datasets. Upset plots showing the degree to which 600 bp non-overlapping genomic windows are shared between five datasets (baseline null, IFNA, dex, HepG2, and Lea *et al.*, 2018; all datasets were analyzed following the same pipeline). Note that the same input library was used in Lea *et al.*, 2018 and the HepG2 experiment, which differs from the library used here for baseline, dex, and IFNA experiments.

bioRxiv preprint doi: https://doi.org/10.1101/2023.05.19.541437; this version posted December 15, 2023. The copyright holder for this preprint (which was not certified by peer review) is the author/funder, who has granted bioRxiv a license to display the preprint in perpetuity. It is made available under aCC-BY 4.0 International license.

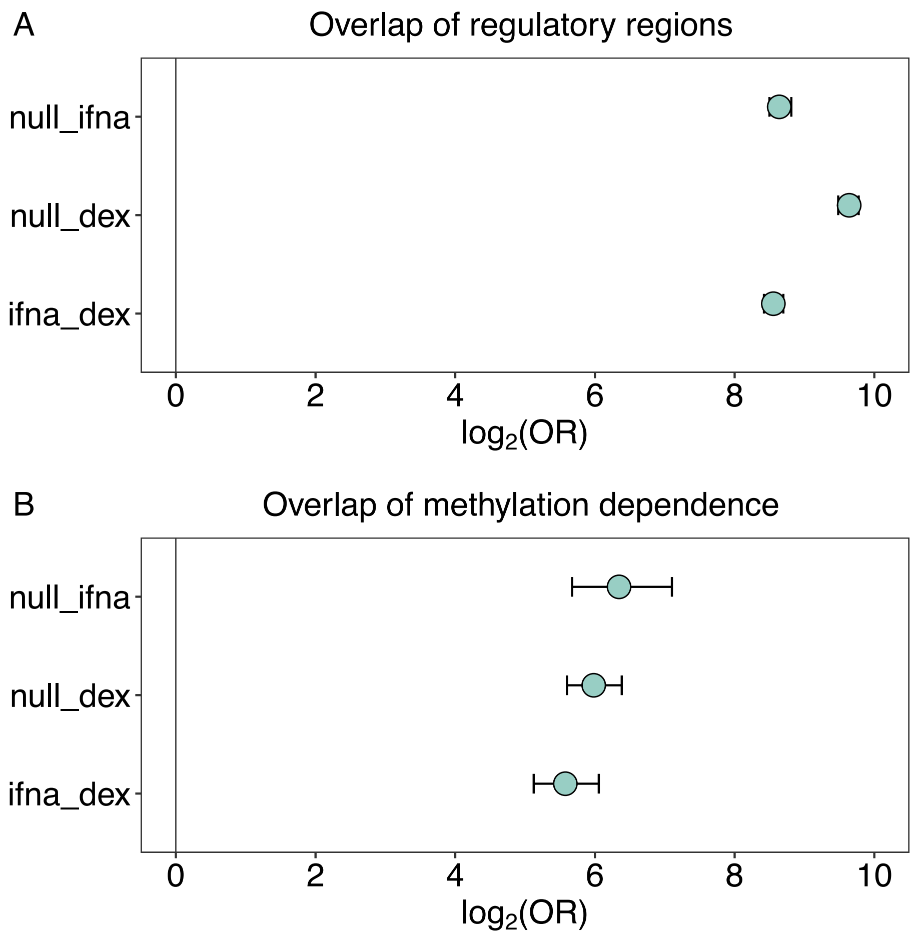

Figure 2-figure supplement 3. Overlap of regulatory activity and effects of methylation across environmental conditions. (A) Regulatory regions in the baseline condition are highly likely to retain regulatory activity upon challenge with IFNA or dex (IFNA log_2(OR) [95% CI] = 8.639 [8.499, 8.812], $p < 1.0 \times 10^{-300}$; dex log_2(OR) [95% CI] = 9.640 [9.483, 9.776], $p < 1.0 \times 10^{-300}$). Regulatory regions also significantly overlap between IFNA- and dex-challenged cells (log_2(OR) [95% CI] = 8.554 [8.420, 8.698], $p < 1.0 \times 10^{-300}$). **(B)** Regulatory windows identified in two environmental conditions tend to share significant effects of DNA methylation on regulatory activity (i.e., interaction effects between methylation and regulatory activity) across the two environmental conditions (baseline and IFNA log_2(OR) [95% CI] = 6.345 [5.673, 7.102], $p < 7.33 \times 10^{-239}$; baseline and dex log_2(OR) [95% CI] = 5.982 [5.599, 6.384], $p < 1.0 \times 10^{-300}$; IFNA and dex log_2(OR) [95% CI] = 5.576 [5.123, 6.055], $p < 4.70 \times 10^{-266}$).

bioRxiv preprint doi: https://doi.org/10.1101/2023.05.19.541437; this version posted December 15, 2023. The copyright holder for this preprint (which was not certified by peer review) is the author/funder, who has granted bioRxiv a license to display the preprint in perpetuity. It is made available under aCC-BY 4.0 International license.

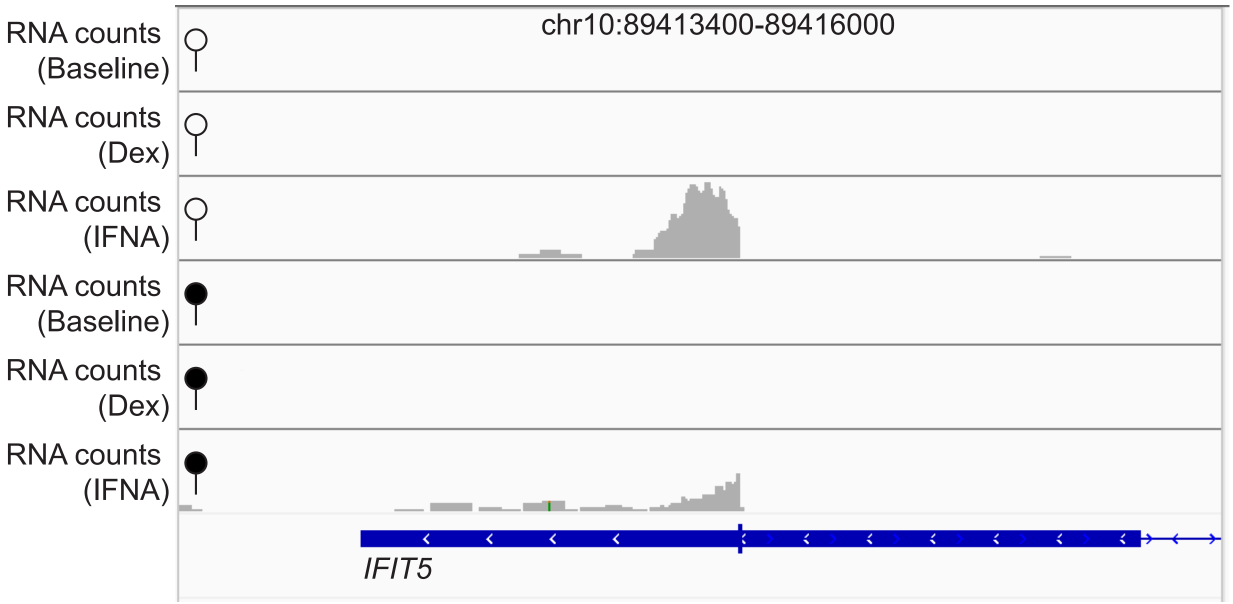

Figure 2-figure supplement 4. *IFIT5* endogenous gene expression is responsive to IFNA stimulation. Tracks show non-normalized, raw read pile-ups of endogenous *IFIT5* (ENSG00000152778) gene expression in either the unmethylated (open circle) or methylated (filled circle) condition, with all y-axis maximums set to 100. The replicates for endogenous gene expression shown here are the same replicates used for measuring regulatory activity shown in Figure 2A. *IFIT5* is only detectably expressed after IFNA stimulation (note that the difference in peak heights between the IFNA-stimulated unmethylated and methylated conditions is because the plot shows raw reads; there is no effect of methylation treatment on endogenous *IFIT5* gene expression after normalization for library size: p = 0.489).

bioRxiv preprint doi: https://doi.org/10.1101/2023.05.19.541437; this version posted December 15, 2023. The copyright holder for this preprint (which was not certified by peer review) is the author/funder, who has granted bioRxiv a license to display the preprint in perpetuity. It is made available under aCC-BY 4.0 International license.

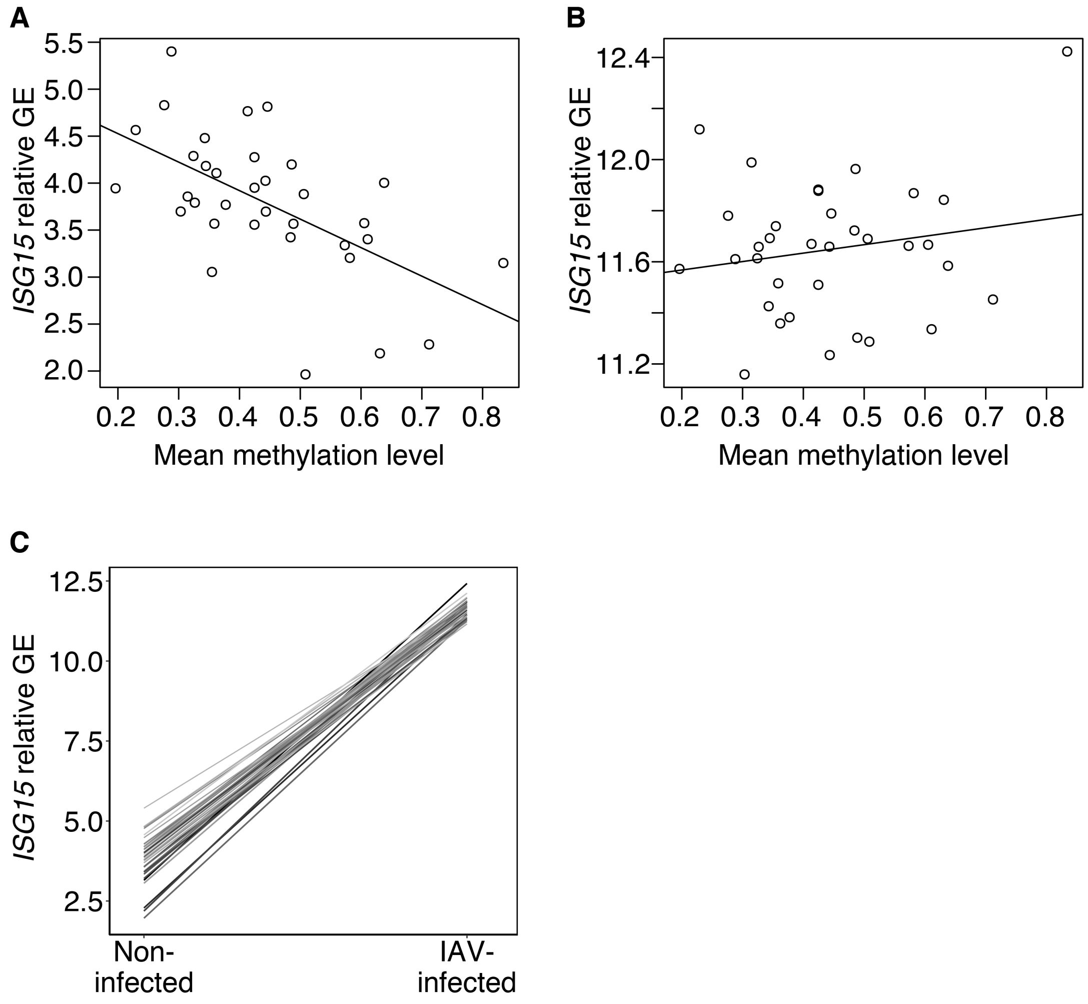

Figure 3-figure supplement 1. Across individuals, methylation in the mSTARR-seq annotated enhancer chr1:1013400-1014000 predicts the *ISG15* gene expression response to flu. (A) Across individuals, average methylation within the mSTARR-seq annotated enhancer chr1:1013400-1014000 in non-infected baseline macrophages significantly predicts *ISG15* gene expression (GE) in the non-infected condition ($R^2 = 0.324$, $p = 2.66 \times 10^{-4}$), but **(B)** not in the IAV-infected condition ($R^2 = 0.001$, $p = 0.316$). Each dot represents an individual. Relative GE is log(CPM) after regressing out the effects of sequencing batch, age, and admixture. **(C)** Individuals with relatively low methylation in the mSTARR-seq chr1:1013400-1014000 enhancer (indicated by lighter line color) in the baseline, non-infected condition tend to have higher *ISG15* gene expression in the non-infected condition, ultimately resulting in a shallower *ISG15* transcriptional response to IAV infection (as indicated by slope of the line). Each line represents an individual.

bioRxiv preprint doi: https://doi.org/10.1101/2023.05.19.541437; this version posted December 15, 2023. The copyright holder for this preprint (which was not certified by peer review) is the author/funder, who has granted bioRxiv a license to display the preprint in perpetuity. It is made available under aCC-BY 4.0 International license.

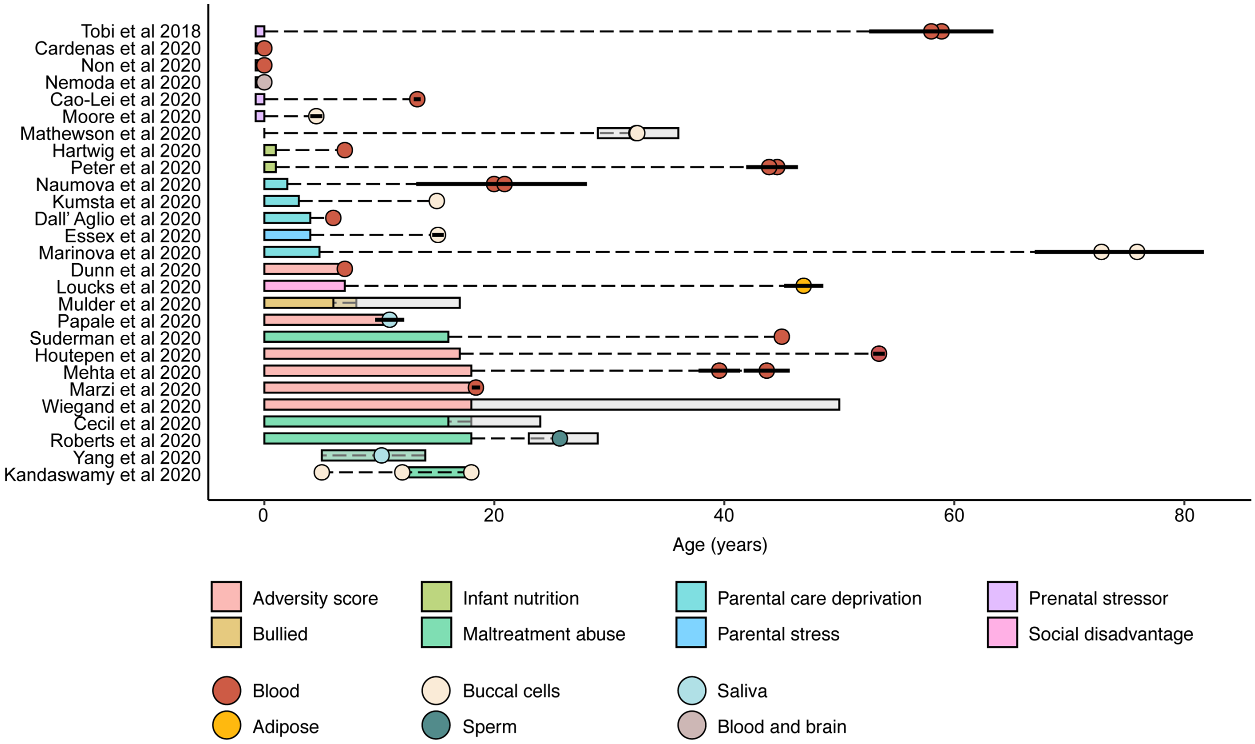

1304 **Figure 4-figure supplement 1. Summary of early-life adversity (ELA) studies.** Colored
1305 rectangles indicate ranges of age at adversity. Circles represent mean ages at sample collection
1306 and are colored according to the tissue type used to measure methylation levels. Ranges and
1307 standard errors of ages at sample collection, when reported, are indicated by gray rectangles and
1308 horizontal black solid lines, respectively. See Supplementary File 20 for results of Fisher's exact
1309 tests assessing enrichment of ELA-associated CpGs for regulatory activity.
