# Supplementary material for: DNA methylation-environment interactions in the human genome
Source: bioRxiv. 2023 May 19:2023.05.19.541437. Preprint. [Version 1] doi: 10.1101/2023.05.19.541437 (PMC10245841; doi:10.1101/2023.05.19.541437)
Supplement: Supplementary file 1 [file NIHPP2023.05.19.541437v1-supplement-18.pdf]

bioRxiv preprint doi: https://doi.org/10.1101/2023.05.19.541437; this version posted May 19, 2023. The copyright holder for this preprint (which was not certified by peer review) is the author/funder, who has granted bioRxiv a license to display the preprint in perpetuity. It is made available under aCC-BY 4.0 International license.

Figure Supplements

Figure 1-figure supplement 1. Overlap of target genomic regions with each other.

bioRxiv preprint doi: https://doi.org/10.1101/2023.05.19.541437; this version posted May 19, 2023. The copyright holder for this preprint (which was not certified by peer review) is the author/funder, who has granted bioRxiv a license to display the preprint in perpetuity. It is made available under aCC-BY 4.0 International license.

Figure 1-figure supplement 2. mSTARR-seq library diversity.

Figure 1-figure supplement 3. Methylation levels of mSTARR-seq DNA, pre- and post-transfection.

Figure 1-figure supplement 4. Overlap of regulatory activity across datasets.

Figure 1-figure supplement 5. Library diversity and regions of regulatory activity in HepG2 cells.

Figure 1-figure supplement 6. Methylation-dependent regulatory activity across datasets.

Figure 2-figure supplement 1. Regulatory activity and effects of methylation across environmental conditions.

Figure 2-figure supplement 2. *IFIT5* endogenous gene expression is responsive to IFNA stimulation.

Figure 3-figure supplement 1. Relationship between baseline methylation in the mSTRR-seq annotated enhancer chr1:1013400-1014000 and *ISG15* gene expression in non-infected and IAV-infected macrophages.

Figure 4-figure supplement 1. Summary of early-life adversity (ELA) studies.

Supplementary Files

Supplementary File 1. Table summarizing the K562 mSTARR-seq DNA and RNA libraries generated in this study.

Supplementary File 2. Table of results from the model testing for methylation-dependent regulatory activity in K562 cells at baseline.

Supplementary File 3. Table of results from the model testing for methylation-dependent regulatory activity in K562 cells at baseline condition using the dataset from (Lea *et al.*, 2018).

Supplementary File 4. Table of results from the model testing for methylation-dependent regulatory activity in HepG2 cells at baseline.

Supplementary File 5. Table of enrichment results of transcription factor binding motifs for regulatory windows with higher activity in the methylated relative to the sham condition.

Supplementary File 6. Table of results from the model testing for methylation-dependent regulatory activity in K562 cells stimulated with IFNA.

Supplementary File 7. Table of results from the model testing for methylation-dependent regulatory activity in K562 cells stimulated with dex.

bioRxiv preprint doi: https://doi.org/10.1101/2023.05.19.541437; this version posted May 19, 2023. The copyright holder for this preprint (which was not certified by peer review) is the author/funder, who has granted bioRxiv a license to display the preprint in perpetuity. It is made available under aCC-BY 4.0 International license.

Supplementary File 8. Table of enrichment results of transcription factor binding motifs for regulatory windows specific to the IFNA treatment (<1% FDR in IFNA and >1% FDR in baseline and Dex).

Supplementary File 9. Table of enrichment results of transcription factor binding motifs for regulatory windows specific to the Dex treatment (<1% FDR in Dex and >1% FDR in baseline and IFNA).

Supplementary File 10. Table of enrichment results of transcription factor binding motifs for regulatory windows specific to the IFNA treatment (<1% FDR in IFNA and >10% FDR in baseline and Dex).

Supplementary File 11. Table of enrichment results of transcription factor binding motifs for regulatory windows specific to the Dex treatment (<1% FDR in Dex and >10% FDR in baseline and IFNA).

Supplementary File 12. Table of results from the model testing for the K562 endogenous gene expression response to IFNA.

Supplementary File 13. Table of Pearson's correlation results, within individuals, between mSTARR-seq enhancer DNA methylation in non-infected macrophages and transcriptional response of nearest genes upon IAV infection.

Supplementary File 14. Table of Pearson's correlation results, within individuals, between mSTARR-seq enhancer DNA methylation in non-infected macrophages and gene expression level in non-infected or IAV-infected macrophages.

Supplementary File 15. Table of Pearson's correlation results, across individuals, between mSTARR-seq enhancer DNA methylation in non-infected macrophages and transcriptional response of nearest genes upon IAV infection.

Supplementary File 16. Table of enrichment results of regulatory activity in early-life adversity associated CpGs relative to regulatory activity in all CpGs on the associated Illumina array.

Supplementary File 17. Library diversity of replicate transformations using the ENCODE Project's PCR bottleneck coefficient (PBC).

bioRxiv preprint doi: https://doi.org/10.1101/2023.05.19.541437; this version posted May 19, 2023. The copyright holder for this preprint (which was not certified by peer review) is the author/funder, who has granted bioRxiv a license to display the preprint in perpetuity. It is made available under aCC-BY 4.0 International license.

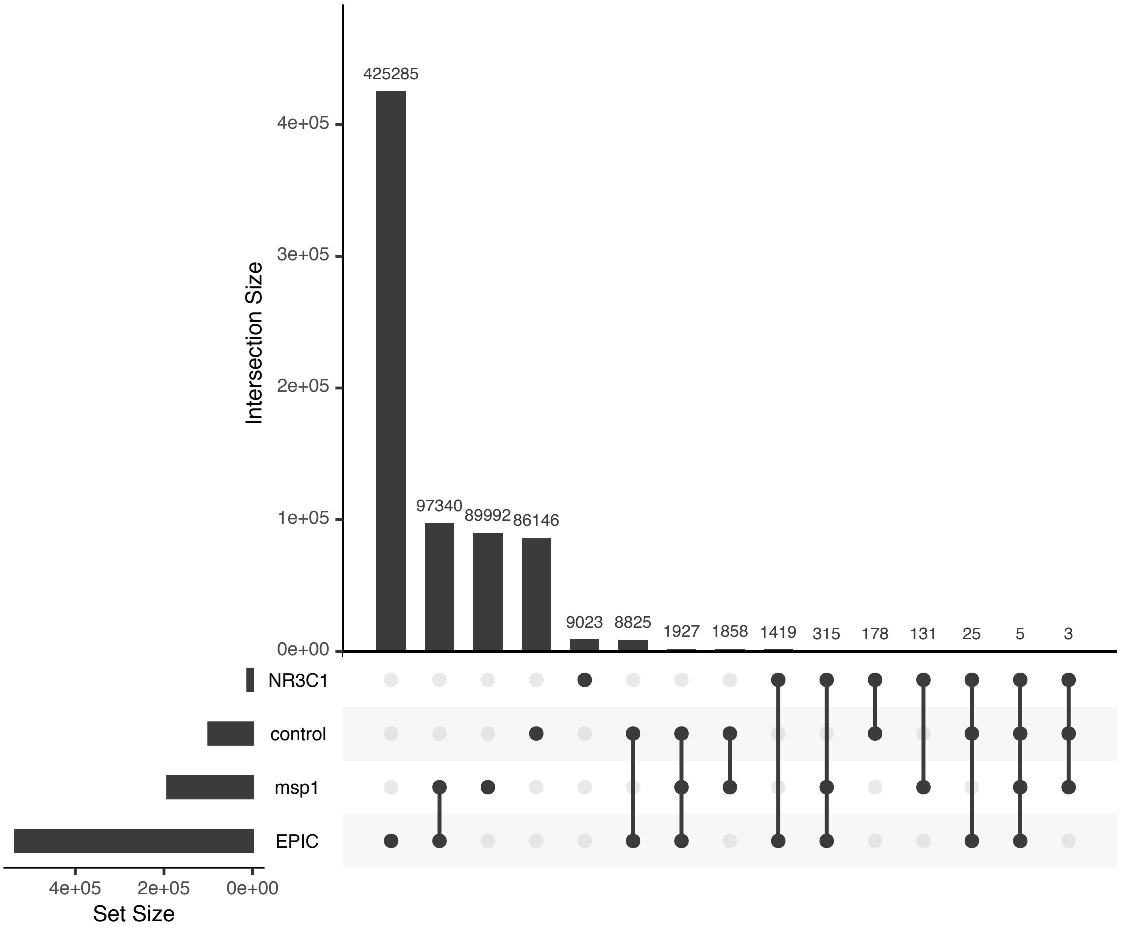

Figure 1-figure supplement 1. Overlap of target genomic regions with each other. Upset plot showing the degree to which 600 bp non-overlapping genomic windows are shared between the four target genomic regions (EPIC CpGs, *Msp1* CpG cut sites, the *NR3C1* region, or control sites). Overlap occurs because a single 600 bp genomic window can simultaneously include EPIC CpGs, *Msp1* CpG cut sites, the *NR3C1* region, and/or control sites. This plot includes 722,472 unique windows, reflecting the set of windows containing at least 1 basepair of target loci.

bioRxiv preprint doi: https://doi.org/10.1101/2023.05.19.541437; this version posted May 19, 2023. The copyright holder for this preprint (which was not certified by peer review) is the author/funder, who has granted bioRxiv a license to display the preprint in perpetuity. It is made available under aCC-BY 4.0 International license.

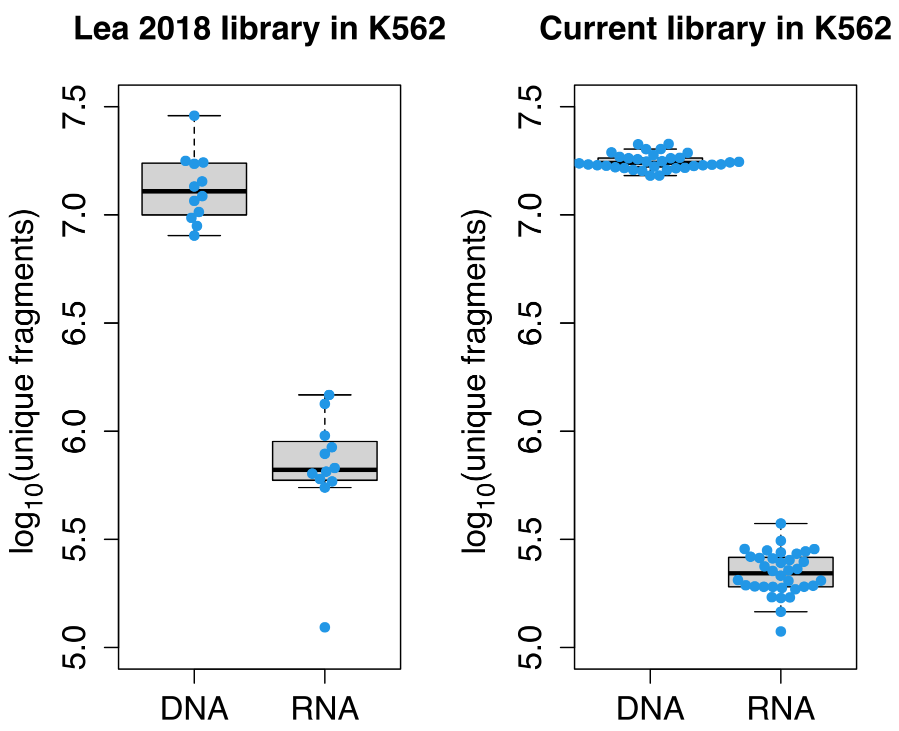

Figure 1-figure supplement 2. mSTARR-seq library diversity. Comparison of diversity of unique mSTARR-seq DNA and RNA fragments from the library generated in this study (transfected into K562 cells) relative to the library published in Lea *et al*., 2018 (independently transfected into K562 cells). Each dot represents an experimental replicate. Each box represents the interquartile range, with the median value depicted as a horizontal bar. Whiskers extend to the most extreme values within 1.5x of the interquartile range.

bioRxiv preprint doi: https://doi.org/10.1101/2023.05.19.541437; this version posted May 19, 2023. The copyright holder for this preprint (which was not certified by peer review) is the author/funder, who has granted bioRxiv a license to display the preprint in perpetuity. It is made available under aCC-BY 4.0 International license.

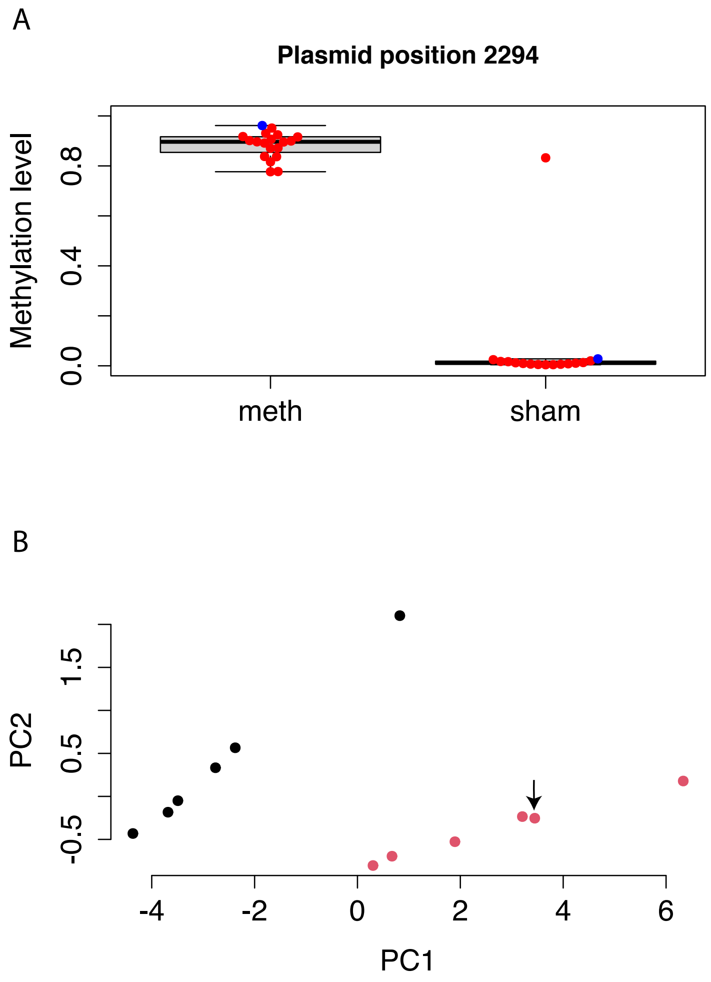

Figure 1-figure supplement 3. Methylation levels of mSTARR-seq DNA, pre- and post-transfection. (A) Bisulfite sequencing shows that DNA methylation on the mSTARR-seq plasmid is maintained until the end of the experiment (i.e., 48 hours after transfection), with significantly higher methylation levels in the replicates from the methyltransferase reaction relative to the replicates from the sham methyltransferase reaction (mean methylated = 0.885, mean unmethylated = 0.066; unpaired t-test: t = -14.66, df = 15,124, p = 2.39 x 10^{-10}). Each dot represents an experimental replicate. Red dots indicate post-transfection DNA samples; the single blue dot per condition indicates pre-transfection DNA methylation levels. Methylation is based on the CpG at the position 2294, which is located in the plasmid region used for Gibson assembly. One sample from the dex sham reaction, L31395, shows an unexpectedly high level of methylation, which appears to be due to an error during generation of the bisulfite sequencing library (e.g., mislabeled tube or poor bisulfite conversion), and not the experimental replicate of cells itself, as the mSTARR-seq RNA library (L31244) from the same replicate clusters with the unmethylated sham replicates as expected (panel B). **(B)** The first two principal components summarizing overall counts of mSTARR-seq reads for the dex-treated RNA samples (i.e., the raw readout of overall regulatory activity). Each dot represents an experimental replicate, with red and black indicating sham and methylated replicates, respectively. Overall regulatory activity of sample L31244 (indicated by arrow) clusters with the sham replicates as expected, suggesting that this replicate was indeed transfected with sham-treated mSTARR-seq DNA.

bioRxiv preprint doi: https://doi.org/10.1101/2023.05.19.541437; this version posted May 19, 2023. The copyright holder for this preprint (which was not certified by peer review) is the author/funder, who has granted bioRxiv a license to display the preprint in perpetuity. It is made available under aCC-BY 4.0 International license.

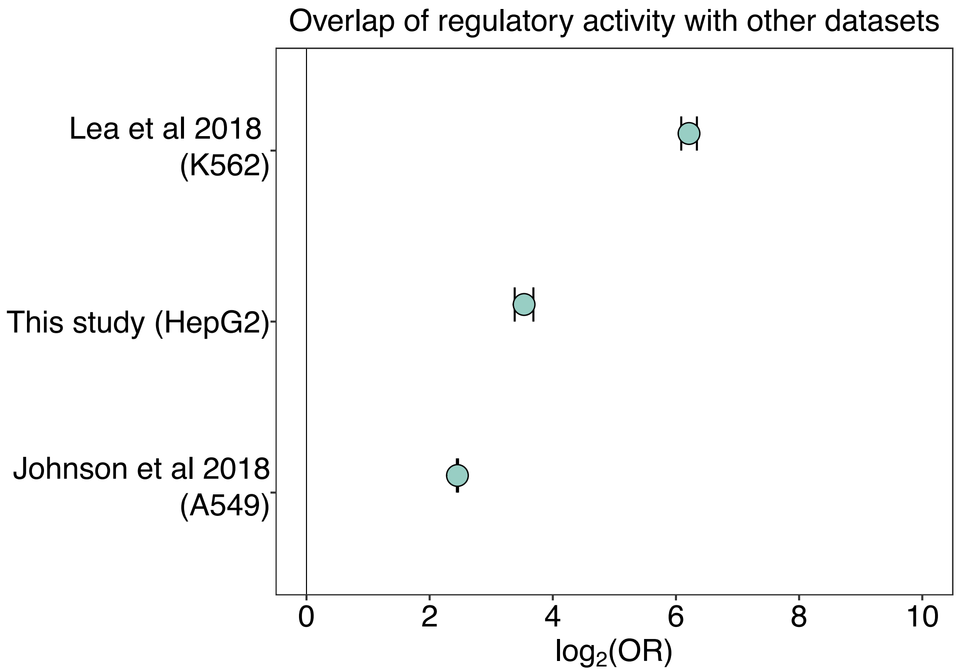

Figure 1-figure supplement 4. Overlap of regulatory activity across datasets. Regulatory regions (in either the unmethylated sham condition, the methylated condition, or both) identified via mSTARR-seq in this study significantly overlap with: K562 regulatory regions (in either the unmethylated sham or methylated condition, or both) from a previously generated mSTARR-seq dataset reanalyzed with our pipeline (Lea et al., 2018) ($\log_2$(OR) [95% CI] = 6.212 [6.086, 6.440], $p < 1.0 \times 10^{-300}$); regulatory regions (in either the unmethylated sham or methylated condition, or both) from an mSTARR-seq experiment in HepG2 liver cells ($\log_2$(OR) [95% CI] = 3.534 [3.381, 3.684], $p = 5.21 \times 10^{-307}$); and regulatory regions from a conventional STARR-seq experiment (i.e., an unmethylated condition) in A549 lung epithelial cells (Johnson et al., 2018) ($\log_2$(OR) [95% CI] = 2.451 [2.442, 2.461], $p < 1.0 \times 10^{-300}$). Bars represent 95% confidence intervals.

bioRxiv preprint doi: https://doi.org/10.1101/2023.05.19.541437; this version posted May 19, 2023. The copyright holder for this preprint (which was not certified by peer review) is the author/funder, who has granted bioRxiv a license to display the preprint in perpetuity. It is made available under aCC-BY 4.0 International license.

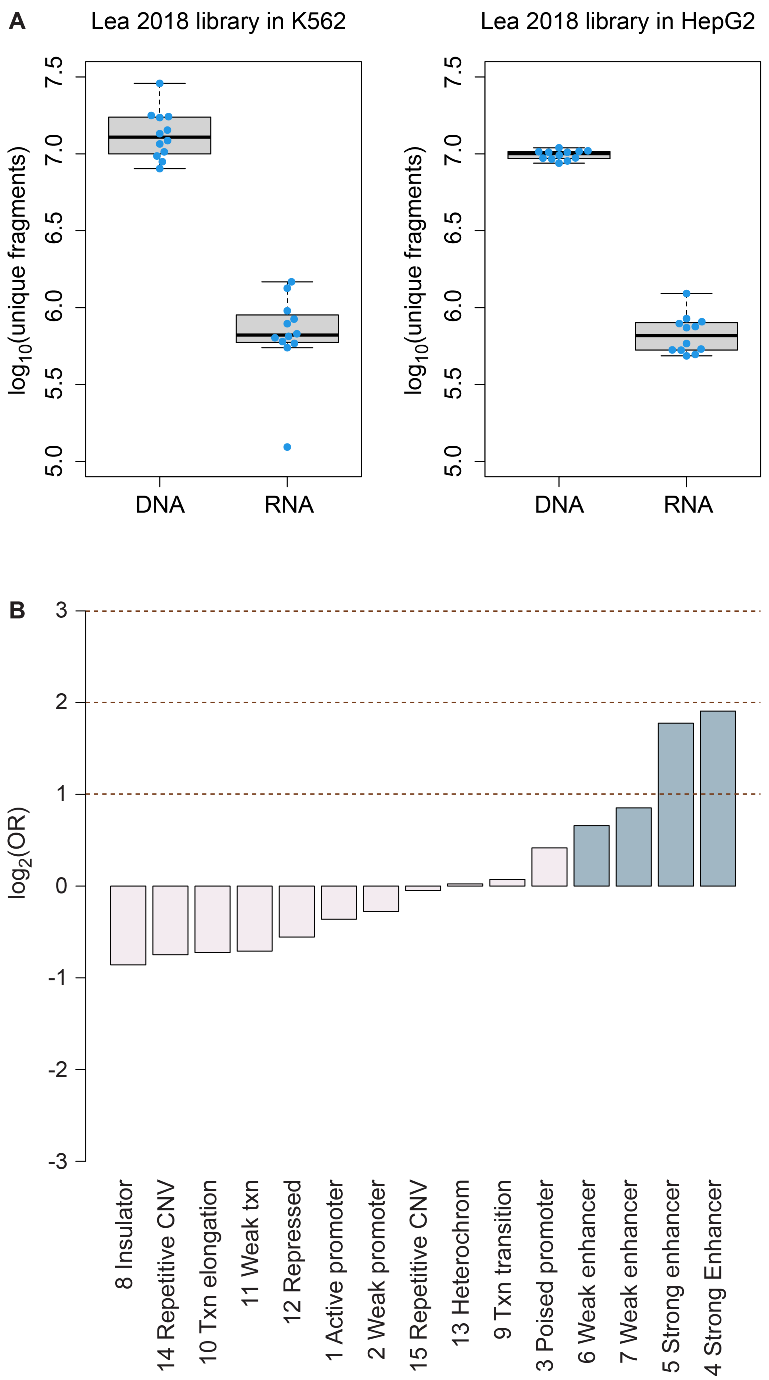

Figure 1-figure supplement 5. Library diversity and regions of regulatory activity in HepG2 cells. (A) Comparison of diversity of unique mSTARR-seq DNA and RNA fragments from the library published in Lea et al 2018 (transfected into K562 cells) versus the same library transfected into HepG2 cells in this study. Each dot represents an experimental replicate. Each box represents the interquartile range, with the median value depicted as a horizontal bar. Whiskers extend to the most extreme values within 1.5x of the interquartile range. **(B)** mSTARR-seq regulatory activity in HepG2 cells is strongly enriched in ENCODE-defined enhancers (indicated in blue) and promoters, and depleted in repressed and repetitive states.

bioRxiv preprint doi: https://doi.org/10.1101/2023.05.19.541437; this version posted May 19, 2023. The copyright holder for this preprint (which was not certified by peer review) is the author/funder, who has granted bioRxiv a license to display the preprint in perpetuity. It is made available under aCC-BY 4.0 International license.

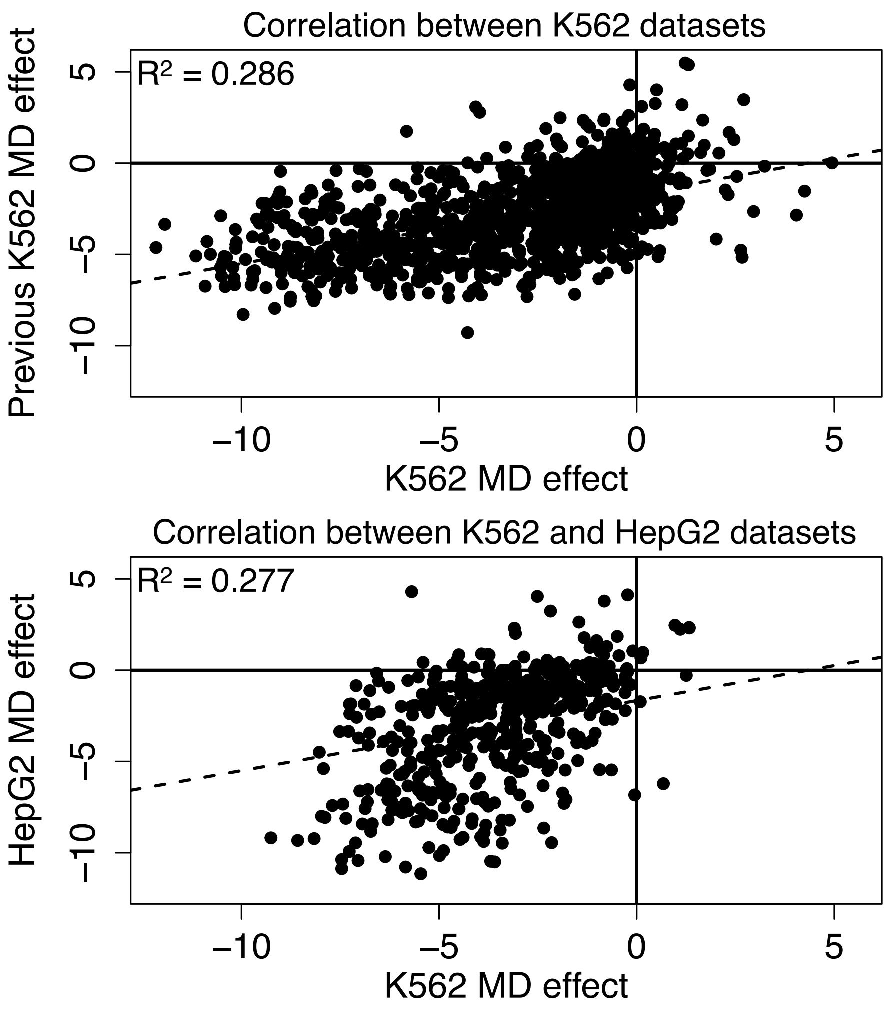

Figure 1-figure supplement 6. Methylation-dependent regulatory activity across datasets. Effects of methylation on regulatory activity estimated in this study are consistent with methylation effects in K562s estimated from a previously generated mSTARR-seq dataset (Lea et al., 2018) and with methylation effects estimated in HepG2 liver cells (K562: $R^2 = 0.286$ for 1250 windows with FDR < 1% in both data sets, $p = 3.19 \times 10^{-93}$; HepG2: $R^2 = 0.277$ for 511 windows with FDR < 1% in both data sets, $p = 8.87 \times 10^{-38}$). Each dot represents a 600 basepair regulatory window identified in both datasets (FDR < 1%; note that not all regulatory windows show significant methylation dependence (MD)). Dashed lines are the best fit lines. In all cases, negative effect sizes correspond to reduced activity in the methylated condition and positive effect sizes correspond to increased activity in the methylated condition.

bioRxiv preprint doi: https://doi.org/10.1101/2023.05.19.541437; this version posted May 19, 2023. The copyright holder for this preprint (which was not certified by peer review) is the author/funder, who has granted bioRxiv a license to display the preprint in perpetuity. It is made available under aCC-BY 4.0 International license.

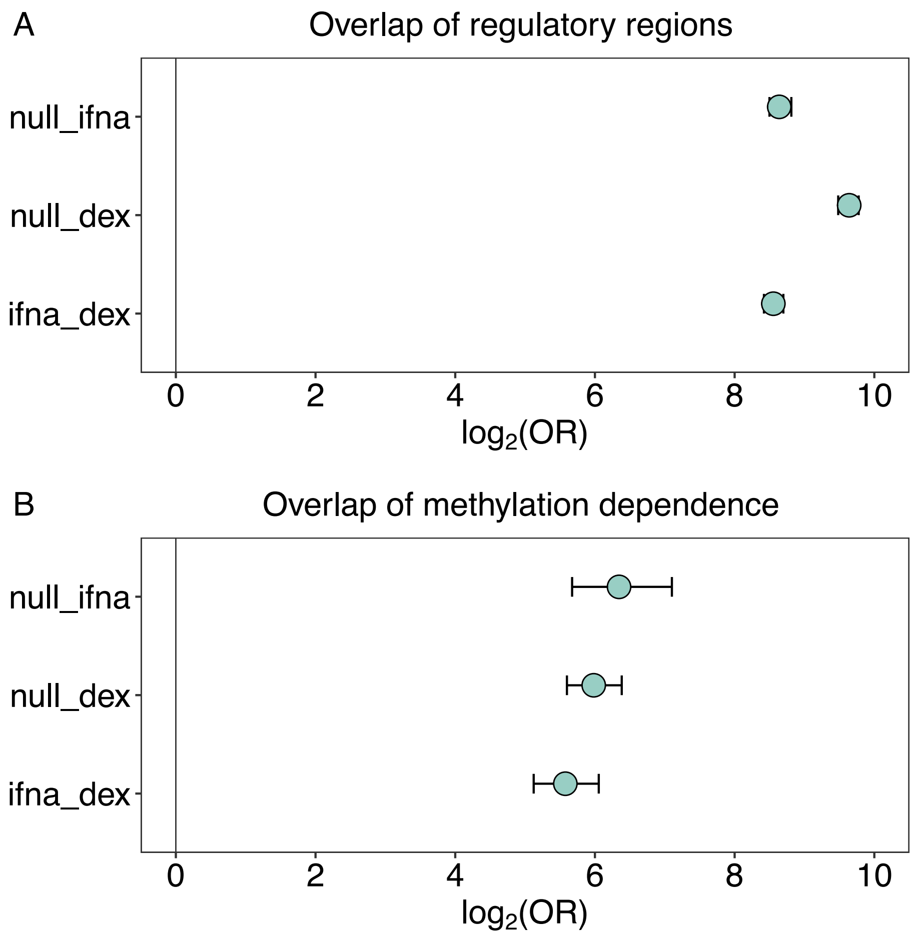

Figure 2-figure supplement 1. Regulatory activity and effects of methylation across environmental conditions. (A) Regulatory regions in the baseline condition are highly likely to retain regulatory activity upon challenge with IFNA or dex (IFNA $\log_2$(OR) [95% CI] = 8.639 [8.499, 8.812], $p < 1.0 \times 10^{-300}$; dex $\log_2$(OR) [95% CI] = 9.640 [9.483, 9.776], $p < 1.0 \times 10^{-300}$). Regulatory regions also significantly overlap between IFNA- and dex-challenged cells ($\log_2$(OR) [95% CI] = 8.554 [8.420, 8.698], $p < 1.0 \times 10^{-300}$). **(B)** Regulatory windows identified in two environmental conditions tend to share significant effects of DNA methylation on regulatory activity (i.e., interaction effects between methylation and regulatory activity) across the two environmental conditions (baseline and IFNA $\log_2$(OR) [95% CI] = 6.345 [5.673, 7.102], $p < 7.33 \times 10^{-239}$; baseline and dex $\log_2$(OR) [95% CI] = 5.982 [5.599, 6.384], $p < 1.0 \times 10^{-300}$; IFNA and dex $\log_2$(OR) [95% CI] = 5.576 [5.123, 6.055], $p < 4.70 \times 10^{-266}$).

bioRxiv preprint doi: https://doi.org/10.1101/2023.05.19.541437; this version posted May 19, 2023. The copyright holder for this preprint (which was not certified by peer review) is the author/funder, who has granted bioRxiv a license to display the preprint in perpetuity. It is made available under aCC-BY 4.0 International license.

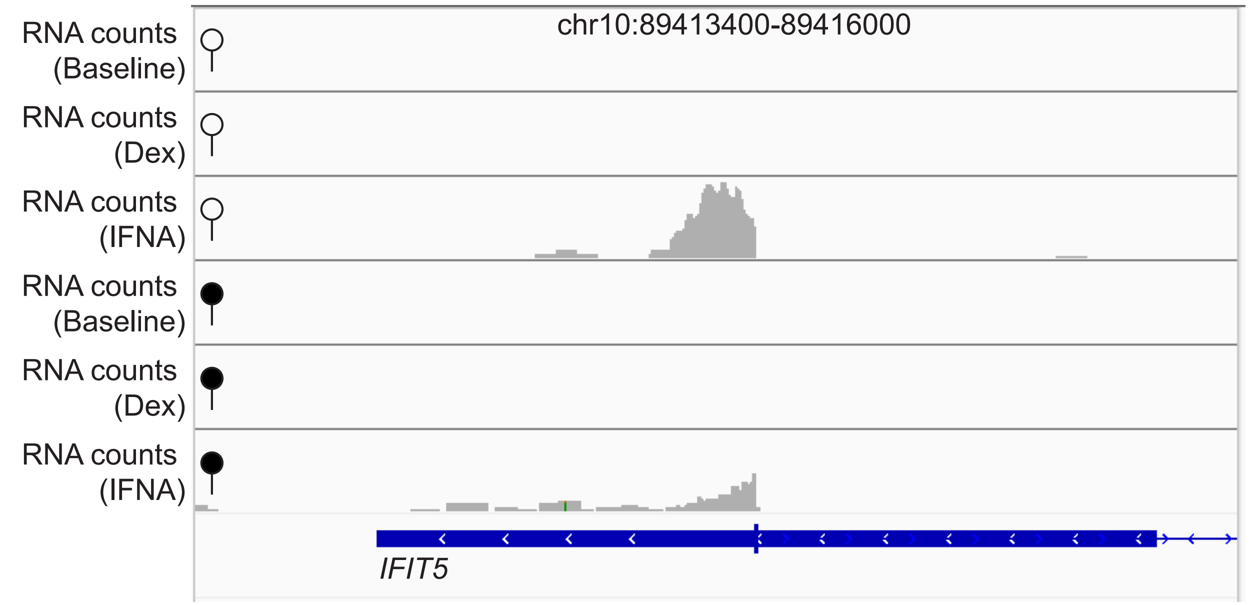

Figure 2-figure supplement 2. *IFIT5* endogenous gene expression is responsive to IFNA stimulation. Tracks show non-normalized, raw read pile-ups of endogenous *IFIT5* (ENSG00000152778) gene expression in either the unmethylated (open circle) or methylated (filled circle) condition, with all y-axis maximums set to 100. The replicates for endogenous gene expression shown here are the same replicates used for measuring regulatory activity shown in Figure 2A. *IFIT5* is only detectably expressed after IFNA stimulation (note that the difference in peak heights between the IFNA-stimulated unmethylated and methylated conditions is because the plot shows raw reads; there is no effect of methylation treatment on endogenous *IFIT5* gene expression after normalization for library size: $p = 0.489$).

bioRxiv preprint doi: https://doi.org/10.1101/2023.05.19.541437; this version posted May 19, 2023. The copyright holder for this preprint (which was not certified by peer review) is the author/funder, who has granted bioRxiv a license to display the preprint in perpetuity. It is made available under aCC-BY 4.0 International license.

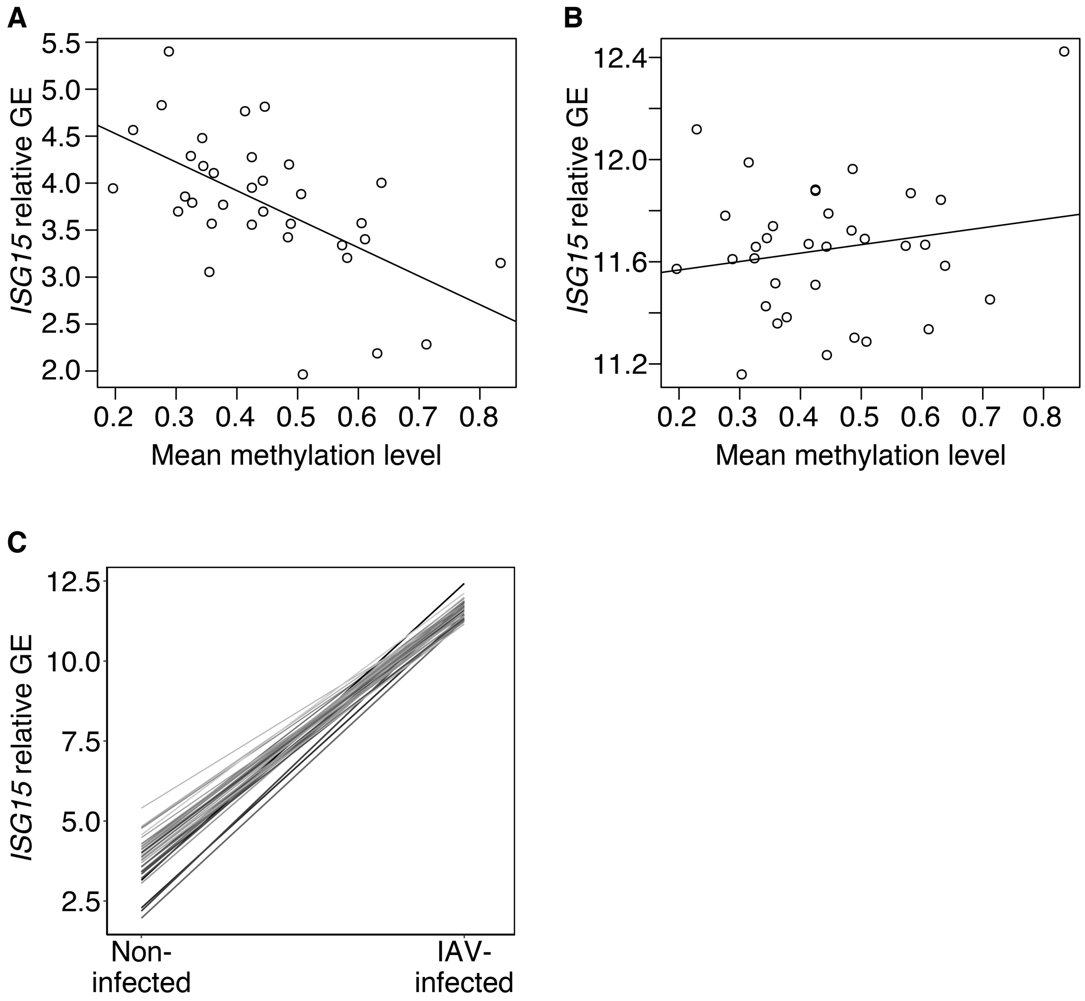

Figure 3-figure supplement 1. Across individuals, methylation in the mSTARR-seq annotated enhancer chr1:1013400-1014000 predicts the *ISG15* gene expression response to flu. (A) Across individuals, average methylation within the mSTARR-seq annotated enhancer chr1:1013400-1014000 in non-infected baseline macrophages significantly predicts *ISG15* gene expression (GE) in the non-infected condition (R^2 = 0.324, p = 2.66 x 10^{-4}), but **(B)** not in the IAV-infected condition (R^2 = 0.001, p = 0.316). Each dot represents an individual. Relative GE is log(CPM) after regressing out the effects of sequencing batch, age, and admixture. **(C)** Individuals with relatively low methylation in the mSTARR-seq chr1:1013400-1014000 enhancer (indicated by lighter line color) in the baseline, non-infected condition tend to have higher *ISG15* gene expression in the non-infected condition, ultimately resulting in a shallower *ISG15* transcriptional response to IAV infection (as indicated by slope of the line). Each line represents an individual.

bioRxiv preprint doi: https://doi.org/10.1101/2023.05.19.541437; this version posted May 19, 2023. The copyright holder for this preprint (which was not certified by peer review) is the author/funder, who has granted bioRxiv a license to display the preprint in perpetuity. It is made available under aCC-BY 4.0 International license.

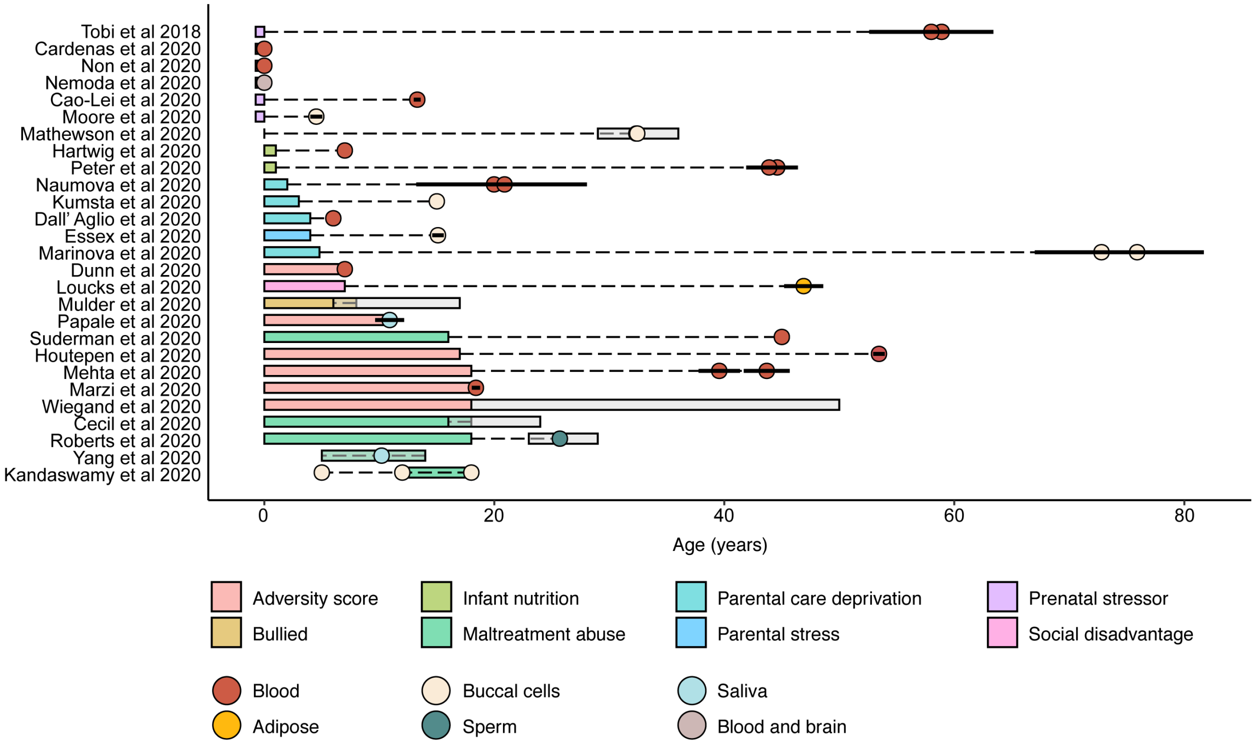

Figure 4-figure supplement 1. Summary of early-life adversity (ELA) studies. Colored and gray rectangles indicate ranges of age at adversity and at sample collection, respectively. Circles represent mean ages at sample collection and are colored according to the tissue type used to measure methylation levels. Black solid line indicates standard errors of ages at sample collection. See Supplementary File 16 for results of Fisher's exact tests assessing enrichment of ELA-associated CpGs for regulatory activity.
